# High-intensity interval training in allogeneic adoptive T-cell immunotherapy – a big HIT?

**DOI:** 10.1186/s12967-020-02301-3

**Published:** 2020-04-01

**Authors:** Nele Carolin Heinemann, Sabine Tischer-Zimmermann, Torge Christian Wittke, Julian Eigendorf, Arno Kerling, Theodor Framke, Anette Melk, Hans-Gert Heuft, Rainer Blasczyk, Britta Maecker-Kolhoff, Britta Eiz-Vesper

**Affiliations:** 1 Institute of Transfusion Medicine and Transplant Engineering, Hannover Medical School, Carl-Neuberg-Straße 1, 30625 Hannover, Germany; 2 Integrated Research and Treatment Center (IFB-Tx), Hannover Medical School, Hannover, Germany; 3 Department of Sports Medicine, Hannover Medical School, Hannover, Germany; 4 Department of Biometry, Hannover Medical School, Hannover, Germany; 5 Department of Pediatric Kidney, Liver and Metabolic Disease, Hannover Medical School, Hannover, Germany; 6 Department of Pediatric Hematology and Oncology, Hannover Medical School, Hannover, Germany

**Keywords:** Virus-specific T cells, T-cell manufacturing, Adoptive T-cell immunotherapy, High-intensity interval training

## Abstract

**Background:**

Adoptive transfer of virus-specific T cells (VSTs) represents a prophylactic and curative approach for opportunistic viral infections and reactivations after transplantation. However, inadequate frequencies of circulating memory VSTs in the T-cell donor’s peripheral blood often result in insufficient enrichment efficiency and purity of the final T-cell product, limiting the effectiveness of this approach.

**Methods:**

This pilot study was designed as a cross-over trial and compared the effect of a single bout (30 min) of high-intensity interval training (HIT) with that of 30 min of continuous exercise (CONT) on the frequency and function of circulating donor VSTs. To this end, we used established immunoassays to examine the donors’ cellular immune status, in particular, with respect to the frequency and specific characteristics of VSTs restricted against Cytomegalovirus (CMV)-, Epstein–Barr-Virus (EBV)- and Adenovirus (AdV)-derived antigens. T-cell function, phenotype, activation and proliferation were examined at different time points before and after exercise to identify the most suitable time for T-cell donation. The clinical applicability was determined by small-scale T-cell enrichment using interferon- (IFN-) γ cytokine secretion assay and virus-derived overlapping peptide pools.

**Results:**

HIT proved to be the most effective exercise program with up to fivefold higher VST response. In general, donors with a moderate fitness level had higher starting and post-exercise frequencies of VSTs than highly fit donors, who showed significantly lower post-exercise increases in VST frequencies. Both exercise programs boosted the number of VSTs against less immunodominant antigens, specifically CMV (IE-1), EBV (EBNA-1) and AdV (Hexon, Penton), compared to VSTs against immunodominant antigens with higher memory T-cell frequencies.

**Conclusion:**

This study demonstrates that exercise before T-cell donation has a beneficial effect on the donor’s cellular immunity with respect to the proportion of circulating functionally active VSTs. We conclude that a single bout of HIT exercise 24 h before T-cell donation can significantly improve manufacturing of clinically applicable VSTs. This simple and economical adjuvant treatment proved to be especially efficient in enhancing virus-specific memory T cells with low precursor frequencies.

## Background

Latent herpes viruses such as Cytomegalovirus (CMV) and Epstein–Barr virus (EBV) as well as lytic viruses such as Adenovirus (AdV) remain an important cause of morbidity and mortality after hematopoietic stem cell transplantation (HSCT) and solid organ transplantation (SOT) in immunocompromised patients [1–3]. With a median of 3 month after HSCT, 11% of all deaths are induced by viral infections with about one-third are caused mainly by CMV (up to 20%), EBV (up to 50%) and AdV (up to 75%) [4–10]. With an incidence of 6 to 28%, AdV infections are the most common infectious complications after HSCT, especially in children [2, 5] Viral complications also influence the outcome of SOT [11] and in general, the incidence varies depending on the transplanted organ, the resulting immunosuppressive treatment, the serostatus of the donor and recipient, and the type of antiviral prophylaxis [12]. Due to high immunosuppression reactivation of EBV and/or outgrowth of latently EBV-infected cells can result in the development of malignant lymphoma (post-transplant lymphoproliferative disease; PTLD), which represents the most common EBV-associated malignancy after transplantation [13].

The pharmacotherapy of viral complications is often limited by both lack of efficacy and toxic side effects. Adoptive transfer of virus-specific T cells has become an attractive option for second line treatment [5, 14–17]. It is established that the delay in the reconstitution of virus-specific CD8^+^ and/or CD4^+^ T-cell responses is a critical factor in viral recrudescence and viral disease and that the permanent control and elimination of a virus depends on the presence of functional virus-specific T cells (VSTs) [18, 19]. The adoptive transfer of donor-derived memory VSTs with defined specificity has developed into a prophylactic and/or curative approach for common post-HSCT and post-SOT viral infections without a significant risk of toxicity or GvHD [2, 3, 20–26]. Recently, we demonstrated the successful use of EBV-specific T cells as a consolidating treatment for a patient suffering from EBV-associated PTLD of the central nervous system [27].

Clinical-grade VSTs can be rapidly isolated directly from blood leukapheresis donations by the cytokine capture system (CliniMACS CCS) [26, 28, 29].

The major challenge is the enrichment of VSTs in sufficient quantities against pathogens with low frequencies of memory VSTs present in the circulation of peripheral blood (e.g. AdV-specific T cells). However, clinical studies showed that the adoptive transfer of even relatively low numbers of VSTs (0.4 × 10^3^ to 8 × 10^4^/kg CMV-VSTs, 0.2 × 10^3^ to 6 × 10^4^/kg EBV-VSTs, 0.3 × 10^3^ to 3 × 10^4^/kg AdV-VSTs) resulted in the decrease in viral load without acute toxicities or GvHD induction [2, 5, 24, 26]. Studies over the last 30 years successfully demonstrated the feasibility of the adoptive T-cell transfer with a treatment response in 74% of 246 evaluable published patients including 85% of CMV-responders, 62% of EBV-responders and 74% of AdV-responders [5, 18, 30–35]. Still, some patients do not respond to VST infusions, possibly because the infusions contain low numbers of VSTs [36].

Methods to increase the number and function of VSTs in healthy donors are an attractive way to ensure the success of adoptive T-cell therapy. As lymphocytosis occurs during and directly after exercise [37], pre-donation exercise could be a time- and cost-efficient way to increase the starting frequencies of circulating peripheral memory VSTs in the donor before apheresis [38–40]. Especially single bouts of exercise evoke increased hemodynamics and the release of catecholamines and glucocorticoids, resulting in a striking leukocytosis [37]. Simultaneously, a redistribution of effector cells between the blood compartment and the lymphoid and peripheral tissues occurs [37]. On the other hand, longer periods of exhaustive exercise can have the adverse effect and weaken immunity [37]. Recent studies have shown that a single bout of continuous exercise (CONT) enhances the quantity of VSTs when they are stimulated and expanded over 8 days [39]. In the study of Kunz et al. they clearly demonstrated that single bouts of exercise can mobilize AdV-specific T cells and increase their function [41]. There is a growing body of evidence showing that high-intensity interval training (HIT) is an effective alternative to traditional endurance training. Alongside similar or even superior effects on many health-related markers (e.g. cardiovascular risk factors), lymphocyte numbers were shown to increase directly after HIT in sedentary men [38–40, 42, 43].

The present study aimed to investigate the benefit of CONT and HIT for adoptive antiviral T-cell therapy. We compared the impact of a 30 min single bout of HIT versus 30 min of CONT on the cellular immune status of healthy donors, in particular, regarding the total number, functionality and proliferative capacity of VSTs. These parameters were analysed in blood samples collected at different time points before and after exercise to identify the time point with the most effective induction level of functional active VSTs and therefore to define the optimal time point for T-cell donation. In summary, we found that both training protocols boost the number and function of VSTs in healthy donors and that the highest response was observed in VSTs against CMV, EBV and AdV with low precursor frequencies in the peripheral blood and in donors with a moderate fitness level who performed HIT 24 h prior to donation.

## Methods

### Study population

After approval by the Institutional Review Board of Hannover Medical School (MHH, approval number 3366-2016), 12 healthy adult volunteers (six females and six males with a mean age of 24 years; range: 20–31 years) were recruited into this study. Their demographic characteristics are presented in Table 1. To be eligible participants had to be over 18 years old, not pregnant and without any evidence of disease. After giving their written informed consent, each participant underwent a medical check-up to exclude active infections and an electrocardiogram in rest was evaluated by an experienced cardiologist to make sure that participants could join the exhaustion test. The participants’ fitness level was assessed using the International Physical Activity Questionnaire (IPAQ) [44] for classification of fitness level. All 12 donors had a moderate (n = 8) or a high (n = 4) fitness level.

**Table 1:** Physical and fitness characteristics of the participants

Characteristics	Group 1 (n = 6)	Group 2 (n = 6)	Total (n = 12)
Sex	3 females, 3 males	3 females, 3 males	6 females, 6 male
Age (years)	22.50 ± 2.07	25.67 ± 4.46	24.08 ± 3.70
Height (cm)	180.33 ± 10.41	175.17 ± 5.15	177.75 ± 8.28
Mass (kg)	77.78 ± 26.34	80.05 ± 13.31	78.92 ± 19.93
BMI (kg/m^2^)	23.44 ± 4.82	26.04 ± 4.09	24.73 ± 4.47
Fitness level (IPAQ-score)	4 moderate, 2 high	4 moderate, 2 high	8 moderate, 4 high
Individual performance limit (W)	221.20 ± 36.27	236.50 ± 41.20	228.83 ± 37.90
Individual performance limit (W/kg)	2.97 ± 0.69	2.9 ± 0.50	2.93 ± 0.57

Each donor executed the two exercise protocols in crossover fashion. At the first visit (week 1), one group (n = 6, group 1) performed a single bout of continuous exercise (CONT) at 50% P_max_ for 30 min, while the other group (n = 6, group 2) performed a single bout of high-intensity interval training (20 min of 30-s 100% P_max_ intervals each followed by a 30-s pause 5 min before and after HIT exercises at 50% P_max_, more precisely High Intensity High Volume Training, HIHVT). At the second visit (week 3), each group switched over to the other exercise protocol. In other words, group 1 did continuous exercise during the first visit now and did HIT exercise on the second (CONT/HIT), and group 2 vice versa (HIT/CONT)

### Exercise protocols and blood sampling

**Fig. 1:**
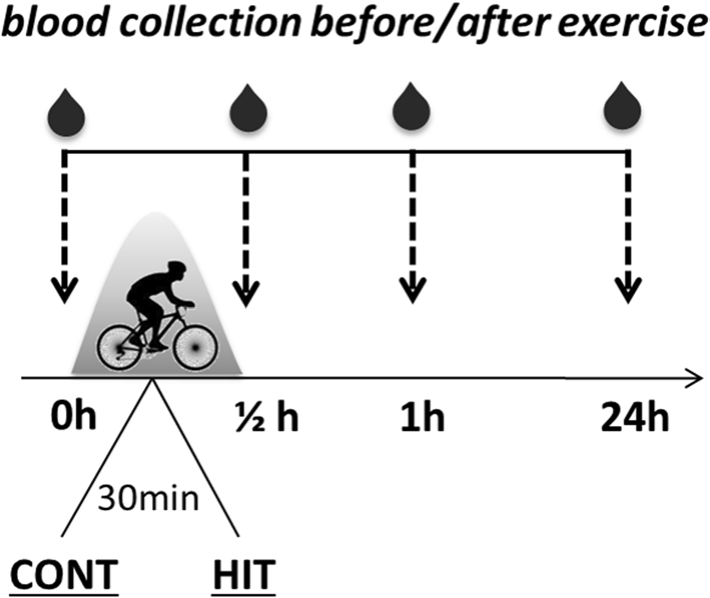
Exercise protocol and blood sampling. Heathy donors (n = 12) were separated into two groups and underwent two different training programs on two occasions separated by 14 days. In the first week group 1 (n = 6) completed a single bout of continuous exercise (CONT: 30 min at 50% of individual maximum capacity), whereas group 2 (n = 6) underwent a single bout of high-intensity interval training (HIT: 20 min of 30 s at maximum capacity followed by a 30 s pause, 5 min before and after HIT at 50% of individual maximum capacity). On the second visit exercise protocols were interchanged for both donor groups (week 3). Intravenous blood samples were collected before, directly, 1 h, and 24 h after exercise. The cellular immune status was determined directly in whole blood and specific T-cell responses were obtained in isolated peripheral blood mononuclear cells (PBMCs) using established immunoassays

Participants were asked to refrain from exercise 24 h prior to each laboratory visit. The individual exercise capacity as measured by maximum power output (P_max_) was determined with a graded exercise test (GXT) on a cycle ergometer (Ergoline, Bitz, Germany) 4 weeks prior to testing. GXT started at 50 W, the workload increased by 16.7 W/min until physical exhaustion. Heart rate (HR) was measured continuously with a 12-channel ECG (GE Electric, Boston USA). Earlobes were hyperaemised at least for 3 min before the first sample was drawn. After 50 W, every 3 min afterwards and at exhaustion capillary blood drawn into 20 µl end-to-end capillaries was used to analyse glucose and lactate concentrations within the next 2 h. Analyses were performed with the Biosen S line analyzer (EKF Diagnostics, Barleben, Germany) including the glucose/lactate test set-up with haemolysing, Glucose/Lactate system, multi standard and ReadyCon test solutions (EKF Diagnostics). On two further occasions separated by a wash-out phase of 14 days, all 12 participants visited the Institute of Sports Medicine at MHH. They were assigned to two equally-sized groups based on the availability of the participants. Blinding was not deemed possible. Each donor executed the two exercise protocols in crossover fashion (Table 1). At the first visit (week 1), one group (n = 6, group 1) performed a single bout of continuous exercise (CONT) at 50% P_max_ for 30 min, while the other group (n = 6, group 2) performed a single bout of high-intensity interval (HIT, more precisely High Intensity High Volume Training, HIHVT) training for 20 min of 30-s 100% P_max_ intervals each followed by a 30-s pause 5 min before and after HIT exercises at 50% P_max_. At the second visit (week 3), each group switched over to the other exercise protocol. Heart rate was measured continuously throughout each visit with a 3-channel ECG (Ergoline, Bitz, Germany), and earlobe capillary blood samples for lactate and glucose testing were drawn before, during and after exercise. Intravenous blood samples were collected from a cubital or antecubital vein before and directly after exercise as well as 1 h and 24 h after exercise (Fig. 1). Complete blood counts and cellular immune status tests were performed immediately on whole blood samples. Peripheral blood mononuclear cells (PBMCs) were isolated by discontinuous density gradient centrifugation and examined for the specified VST characteristics using appropriate in vitro immunoassays. Representative flow cytometric plots of the cellular immune status (A), VST enrichment efficiency (B) and T-cell activation using CD69 as a specific marker (C) are visualized in Additional file 1.

### Serological testing by ELISA and Western blot

All donors were pretested for CMV, EBV and AdV serostatus as described previously using commercially available IgG Western blot and IgG ELISA kits [45, 46]. Briefly, CMV and EBV serology was performed by confirmatory Western blot tests designed to quantitatively determine anti-CMV or anti-EBV IgG antibodies against major CMV and EBV proteins (recomLine CMV or EBV IgG, Mikrogen, Neuried, Germany). IgG antibodies against AdV were detected using the alphaWell Adenovirus IgG ELISA (Mikrogen) according to the manufacturer’s instructions.

### Determination of the frequencies of antiviral memory T cells by enzyme-linked immunospot assay

Detection of virus-specific IFN-γ-producing T-lymphocytes was achieved by IFN-γ EliSpot assay as previously described. Briefly, PBMCs were isolated from whole blood samples by discontinuous density gradient centrifugation, resuspended in culture medium (CM) consisting of RPMI1640 (Lonza, Vervies, Belgium) with 10% human AB serum (C.C.pro, Oberdorla, Germany) at a concentration of 1 × 10^7^ cells/ml, seeded on 24-well plates and rested overnight. Rested PBMCs were co-cultured in anti-IFN-γ precoated EliSpot plates (Lophius Biosciences, Regensburg, Germany) for 16–18 h at a density of 2.5 × 10^5^ cells/well with specific antigens of interest. For stimulation CMV-derived peptide pools CMV pp65 and CMV IE-1, EBV-derived peptide pools EBV EBNA1 and EBV Consensus and AdV-derived peptide pools AdV5 Hexon and AdV5 Penton were used at a final concentration of 1 µg of each peptide/ml peptide pool (Miltenyi Biotec, Bergisch Gladbach, Germany). Cells stimulated with staphylococcal enterotoxin B (1 µg/ml, SEB, Merck, Taufkirchen, Germany) served as the positive control and PBMCs incubated in media alone as the negative control. IFN-γ secretion was detected using streptavidin–alkaline phosphatase (Mabtech Stockholm, Sweden) and revealed by 5-13 bromo-4-chloro-3-indolyl phosphate/nitroblue tetrazolium (BCIP/NBT Liquid Substrate, Merck). Spots were counted using AID EliSpot 8.0 on an AID iSpot spectrum reader system (both from AID, Strassberg, Germany). Findings are indicated as number of spots per well (spw), representing the number of spots in the antigen well after subtracting the respective negative control well. Results were furthermore indicates as spw/1000 CD3^+^ T cells.

### IFN-γ Cytokine Secretion Assay

The IFN-γ Cytokine Secretion Assays (CSA, Miltenyi Biotec) was performed as previously described [47]. After overnight resting, 1 × 10^7^ isolated PBMCs were stimulated with the CMV peptide pools CMV pp65 or CMV IE1, EBV peptide pools EBV EBNA1 or EBV Consensus, and AdV peptide pools AdV5 Hexon or AdV5 Penton, respectively, at a final concentration of 1 µg of each peptide/ml peptide pool. Unstimulated PBMCs served as the negative control. Activated IFN-γ-secreting T cells were specifically captured during the magnetic cell sorting enrichment process by using anti-IFN-γ-phycoerythrin (PE) antibodies and paramagnetic anti-PE microbeads. Aliquots of the respective cell fractions collected before and after enrichment were used for detailed analysis of IFN-γ^+^ T-cell subsets by multicolour flow cytometry. The distribution of viable and dead cells in these fractions was analysed by 7AAD (7-amino-actinomycin D) staining (BD Biosciences, Heidelberg, Germany). The percentage of viable IFN-γ^+^ cells was determined by staining the cells with anti-CD45-allophycocyanin plus cyanin-7 (APC/Cy7), anti-CD3-fluorescein isothiocyanate (FITC), anti-CD8-APC, and anti-CD4-Alexa-Fluor 700) monoclonal antibodies (mAbs, all from BD Biosciences). At least 10,000 events were acquired in the viable CD45^+^ leukocyte gate for each analysis (FACSCanto10c, BD Biosciences). CD3^+^/IFN-γ^+^, CD8^+^/IFN-γ^+^ and CD4^+^/IFN-γ^+^ T-cell populations were gated based on the scatter properties of viable 7AAD^−^/CD45^+^/CD3^+^ T lymphocytes. Results are indicated as frequencies (in %) of IFN-γ^+^ T cells after subtracting the respective negative control.

### Detection of the cellular immune status by flow cytometry

All flow cytometric analyses were performed using the FACSCanto 10c system (BD Biosciences) and BD FACSDiva Software version 8.0.1. The cellular immune status was determined by using specific markers for monocytes, T cells, B cells and natural killer (NK) cells. Briefly, whole blood samples were analysed on a single-cell platform using TruCount tubes (BD Biosciences). Absolute counts of the investigated leukocyte subsets were calculated according to the manufacturer’s instructions. Cells were stained for 20 min at room temperature using anti-CD45 APC-H7, anti-CD3 FITC, CD8 APC, anti-CD4 peridinin chlorophyll protein (PerCP), anti-CD19 PE-Cy7, anti-CD56 PE and anti-CD14 BV510 mAbs (all from BD Biosciences) before the lysis of erythrocytes using 1× BD FACS Lysing Solution according to the manufacturer’s instructions (BD Biosciences). The number of spots per 1000 CD3^+^ T cells, as determined by IFN-γ EliSpot assay, was calculated based on the CD3 frequencies determined by staining PBMCs with anti-CD45 APC-H7, anti-CD3 FITC, anti-CD8 APC and anti-CD4 PerCP).

### Real-time PCR detection of HSP70, Ki67, IFN-γ, and granzyme B expression measurement of telomere length

The mRNA levels of heat shock protein 70 (HSP70), Ki67, IFN-γ, and granzyme B were analysed as previously described [48]. Briefly, total cellular RNA was isolated (RNeasy Mini Kit; Qiagen, Hilden, Germany), and cDNA was amplified using the High Capacity cDNA Reverse Transcription Kit (Applied Biosystems, Darmstadt, Germany). Quantification of HSP70, Ki67, IFN-γ, and granzyme B mRNA levels was achieved by using inventoried mixes (Applied Biosystems). TaqMan Gene Expression Master Mix (Applied Biosystems) was used for amplification. The constitutively expressed glyceraldehyde 3-phosphate dehydrogenase (GAPDH) gene served as the reference gene.

As an indicator of telomere length, p16 expression was analysed. Quantitative PCR of p16INK4a was performed using the following intron-spanning primers and probes: p16INK4a forward 5′-GGGCACTGCTGGAAGCC-3′, reverse 5′-AACGTTGCCCATCATCATC-3′, and probe 5′-CCGAACTCTTTCGGTCGTA-3′. For analysis of telomere length, genomic DNA was first extracted from isolated PBMCs using the QIAamp DNA Mini Kit (Qiagen, Hilden, Germany). DNA quantification was then performed on an Infinite M200 Pro microplate reader (Tecan, Maennedorf, Switzerland) using SYBR^®^Green I Dye (Invitrogen, Carlsbad, CA, USA). Telomere length was calculated as abundance of telomeric template versus a single copy gene (36B4) by quantitative real-time PCR (qPCR) on a 7900HT Fast Real-Time PCR System (Applied Biosystems, Darmstadt, Germany) as previously described [49, 50]. Results are expressed as the ratio of the telomere repeat copy number to the single gene copy number (T/S). All measurements were performed in quadruplicate, and all samples were measured on a single plate to reduce inter-assay variability.

### Quantification of the total cellular cholesterol level of cytotoxic CD8^+^ T cells

CD8^+^ T cells were isolated from blood samples collected at different time points directly before and after exercise (Whole Blood CD8 MicroBeads, Miltenyi Biotec) for quantification of total cellular cholesterol. The cells were fixed with 0.1% glutaraldehyde (Merck, Taufkirchen, Germany) and treated with 2 U/ml cholesterol oxidase (ThermoFisher) for 15 min to oxidize the plasma membrane cholesterol. After extracting intracellular cholesterol with methanol/chloroform (vol/vol, 1:2), total cellular cholesterol was quantified using the Amplex Red Cholesterol Assay Kit (ThermoFisher) according to the manufacturer’s instructions. Plasma membrane cholesterol levels were calculated by subtracting intracellular cholesterol from total cellular cholesterol. The purity of isolated CD8^+^ T cells was assessed by flow cytometry after staining the cells with anti-CD8 APC mAb (BD Biosciences). At least 10,000 events were acquired in the CD8^+^ T-cell gate. The percentage of activated CD8^+^ T cells was determined by staining the cells with anti-CD3 PerCP, anti-CD8 APC, and anti-CD69 FITC mAbs (all from BD Biosciences). At least 20,000 events were acquired in the CD3^+^ T-cell gate for each analysis, while CD69^+^ T cells were gated on the scatter properties of CD3^+^/CD8^+^ T lymphocytes.

### Statistical analysis

This pilot study was carried out as a non-randomized two period two sequences cross-over trial. We did not adjust for multiplicity due to the exploratory nature of this trial and hence, our results are not to be interpreted in a confirmatory sense.

Statistical analysis was performed using the Prism v5.02 software (GraphPad, San Diego, California, USA) and SAS 9.4 (SAS Institute Inc., Cary, NC, USA). The results are displayed as mean ± standard deviation (SD). Data were analysed using the Wilcoxon test to compare each post training measurement with the pre-training measurement separately. Significance levels were calculated and expressed as p-values (*p < 0.05, **p < 0.01, ***p < 0.001). In addition, we carried out calculations to compare the two training methods with the two sample t approach adjusting for period as described in chapter 3 of [51].

## Results

### Exercise induced lymphocytosis

All donors successfully completed the two 30 min exercise sessions. The effects of both protocols on the standard complete blood count (CBC) and cellular immune status were investigated with respect to changes in heart rate, lactate and glucose levels (Table 2) as well as to leukocyte subsets, erythrocytes and platelets (Table 3, Additional file 2). Heart rate was increased significantly by both programs during and directly after exercise, while glucose levels decreased. The measured lactate levels were higher during exercise and began to decrease again directly after. Both CONT (6.48 × 10^3^/µl ± 1.38 before exercise vs. 8.242 ± 1.50 1 h after exercise, p < 0.01) and HIT (6.72 × 10^3^/µl ± 1.19 before exercise vs. 8.36 × 10^3^/µl ± 2.32 1 h after exercise) increased the total count of leukocytes (white blood cells (WBCs)) in the standard CBC (Table 3, Panel A). All investigated immune cell subsets analysed in blood samples by using the haemocytometer, namely lymphocytes, granulocytes and monocytes (Table 3, Panel A, Additional file 2), indicated an increase of the absolute cell numbers 1 h after exercise, with recovery at least after 24 h. There was no significant difference between both training programs. Concerning the leukocytes, we found a higher increase of granulocytes compared to lymphocytes. This was confirmed by a slight decrease of the absolute lymphocyte count compared to an increase of the absolute leukocytes count (Table 3, Panel A). Similar results were obtained by single-cell flow cytometric analysis, investigating the frequency and absolute number of CD45^+^ leukocyte and lymphocyte subsets (Table 3, Panel B; Additional file 2 and Additional file 3, shown as normalized data). Interestingly, the absolute number of CD3^+^ T cells was increased continuously up to 24 h after exercise (Additional file 2E, shown as normalized data), while the CD4/CD8 T-cell ratio increased only slightly within 1 h after both training protocols and almost completely returned to pre-exercise levels within 24 h post training (Additional file 2F, shown as normalized data).

**Table 2:** Blood glucose, blood lactate, and heart rate in response to exercise

Parameter	Program	Before exercise	During exercise	After exercise
Glucose (mmol/l)	CONT	4.48 ± 0.40	4.15 ± 0.47	3.97 ± 0.41**
	HIT	5.12 ± 0.72	4.49 ± 0.50*	4.35 ± 0.46*
Lactate (mmol/l)	CONT	1.00 ± 0.37	3.31 ± 1.21***	2.91 ± 1.61**
	HIT	0.97 ± 0.18	6.12 ± 1.98***	5.82 ± 2.54***
Heart rate (bpm)	CONT	135.69 ± 15.00	153.07 ± 18.96*	159.11 ± 17.75*
	HIT	138.66 ± 13.59	162.22 ± 14.61**	165.40 ± 15.66**

Glucose and lactate levels in peripheral blood and heart rate before, during and after exercise (CONT: continuous exercise, HIT: high-intensity interval training). Glucose and Lactate measured periodically, heart rate measured continuously. Asterisks indicate statistically significant differences between before, during and after exercise (*p < 0.05, **p < 0.01 and ***p < 0.001). Data are mean ± SD

**Table 3:** Total and differential leukocyte counts, total red blood cell and platelet counts

Parameter	Program	Before exercise	After exercise	1 h after exercise	24 h after exercise
Panel A					
Leukocytes (×10^3^/µl]	CONT	6.48 ± 1.38	7.59 ± 1.52	8.24 ± 1.50**	7.02 ± 1.74
	HIT	6.72 ± 1.19	7.51 ± 2.08	8.36 ± 2.32	6.87 ± 1.15
Lymphocytes (%)	CONT	33.13 ± 8.10	31.97 ± 8.35	22.30 ± 6.58**	28.86 ± 8.86
	HIT	33.18 ± 8.40	30.14 ± 5.70	25.48 ± 6.84**	31.37 ± 7.98
Monocytes (%)	CONT	7.68 ± 8.1	7.59 ± 1.13	6.25 ± 1.42*	6.97 ± 1.64
	HIT	7.64 ± 1.74	7.57 ± 1.42	6.95 ± 1.03	7.58 ± 1.04
Granulocytes (%)	CONT	59.18 ± 8.17	60.28 ± 8.55	71.45 ± 7.15**	64.18 ± 9.43
	HIT	59.18 ± 8.98	61.84 ± 5.32	67.55 ± 7.42*	61.03 ± 8.62
Lymphocytes (×10^3^/µl)	CONT	2.06 ± 0.39	2.38 ± 0.50	1.80 ± 0.47	1.81 ± 0.66
	HIT	2.18 ± 0.54	2.21 ± 0.61	2.07 ± 0.52	2.09 ± 0.51
Monocytes (×10^3^/µl)	CONT	0.53 ± 0.14	0.58 ± 0.10	0.52 ± 0.13	0.48 ± 0.11
	HIT	0.52 ± 0.17	0.58 ± 0.20	0.59 ± 0.17	0.53 ± 0.11
Granulocytes (×10^3^/µl)	CONT	3.88 ± 1.19	4.64 ± 1.36	5.93 ± 1.46**	4.63 ± 1.73
	HIT	3.99 ± 1.02	4.73 ± 1.43	5.75 ± 1.92*	4.17 ± 0.98
Red blood cell count (×10^6^/µl)	CONT	5.25 ± 0.94	5.30 ± 0.64	5.26 ± 0.99	5.299 ± 1.09
	HIT	5.37 ± 0.64	5.44 ± 0.82	5.15 ± 0.91	5.028 ± 0.90
Hemoglobin [g/dl)	CONT	15.36 ± 2.85	15.44 ± 1.97	15.32 ± 2.95	15.50 ± 3.24
	HIT	15.69 ± 2.49	15.78 ± 2.30	14.96 ± 2.59	14.63 ± 2.55
Hematocrit [%)	CONT	46.84 ± 8.41	47.00 ± 5.54	46.48 ± 8.49	46.92 ± 9.38
	HIT	47.81 ± 6.43	47.99 ± 6.78	45.41 ± 7.69	44.43 ± 7.56
Platelet count (×10^3^/µl)	CONT	185.80 ± 67.21	213.20 ± 75.70	187.30 ± 72.87	176.30 ± 72.45
	HIT	185.10 ± 48.55	203.40 ± 65.69	178.80 ± 53.26	186.80 ± 54.98
Plateletcrit (%)	CONT	0.17 ± 0.05	0.19 ± 0.06	0.16 ± 0.06	0.15 ± 0.06
	HIT	0.17 ± 0.05	0.18 ± 0.06	0.16 ± 0.05	0.16 ± 0.05

Parameter	Program	Before exercise	After exercise	1 h after exercise	24 h after exercise
Panel B					
CD45^+^ Leukocytes (/µl)	CONT				
	Mean	1.12E+04	1.26E+04	1.33E+04	1.20E+04
	SD	1.48E+04	1.81E+04	1.91E+04	2.26E+04
	HIT				
	Mean	9.37E+03	1.19E+04	1.31E+04	6.20E+03
	SD	8.50E+03	1.44E+04	1.58E+04	9.41E+02
CD45^+^ Lymphocytes (%)	CONT				
	Mean	32.19	32.00	23.63**	34.15
	SD	7.22	7.20	4.82	6.76
	HIT				
	Mean	31.50	29.22	23.84	31.22
	SD	8.08	5.78	6.13	7.14
CD45^+^ Lymphocytes (/µl)	CONT				
	Mean	2.17E+03	2.26E+03	1.88E+03	1.95E+03
	SD	5.90E+02	5.51E+02	4.53E+02	6.28E+02
	HIT				
	Mean	2.09E+03	2.15E+03	1.91E+03	1.93E+03
	SD	5.65E+02	5.83E+02	6.02E+02	5.29E+02
CD3^+^ T cells (%)	CONT				
	Mean	74.97	75.57	78.45	77.74
	SD	5.20	6.51	5.49	5.62
	HIT				
	Mean	75.81	76.29	77.25	77.62
	SD	4.27	5.16	5.29	4.78
CD3^+^ T cells (/µl)	CONT				
	Mean	1.62E+03	1.71E+03	1.48E+03	1.53E+03
	SD	4.34E+02	4.58E+02	3.87E+02	5.38E+02
	HIT				
	Mean	1.58E+03	1.64E+03	1.48E+03	1.50E+03
	SD	4.02E+02	4.53E+02	4.63E+02	4.19E+02

Cell counts in peripheral blood (Panel A) analysed by using a haemocytometer as well as (Panel B) total leukocyte counts, total and proportional lymphocyte and T-cell counts analysed by flow cytometry before, directly after, 1 h after and 24 h after 30 min of continuous (CONT) and high-intensity interval (HIT) cycling exercise (n = 12). Statistically significant difference from before exercise indicated by (*p < 0.05 and **p < 0.01). Data are mean ± SD

No significant differences between both training programs were identified with regards to the frequencies of the investigated cellular immune subsets (CD45^+^ leukocytes, CD45^+^ lymphocytes, CD14^+^ monocytes, CD19^+^ B cells, CD3^+^CD56^+^ NKT cells and CD3^−^CD56^+^ NK^dim/bright^ cells (Additional file 3, shown as normalized data). The frequency of CD14^+^ monocytes, was increased directly after both exercise programs, while the frequency of CD19^+^ B cells increased within 1 h after both exercise programs and recovered within 24 h (Additional file 3, shown as normalized data). NK cell subsets displayed an inversely proportional trend (Additional file 3). The frequency of CD3^−^/CD56^dim^ NK cells increased within 1 h after both types of exercise, while the frequency of CD3^−^/CD56^bright^ NK cells decreased. Regarding donor fitness level, highly fit individuals had slightly higher frequencies of lymphocytes, in the resting state (Additional file 4A), while absolute numbers of white blood cells (WBC), lymphocytes, granulocytes and monocytes did not differ between donors with high and moderate fitness levels (Additional file 4B).

### Exercise increased HSP70 levels while downregulating proliferation markers and not affecting replicative senescence

Quantitative real-time PCR showed that mRNA expression levels of HSP70 increased more than twofold within 24 h after HIT (2.9-fold), but only slightly after CONT exercise (up to 1.8-fold); the latter increases were detected directly and 1 h post exercise (Additional file 5A). The mRNA expression levels of the proliferation marker Ki67 were lower than baseline at all time points after both types of exercise. The mRNA levels of IFN-γ remained unchanged after CONT and increased slightly within 24 h after HIT. Granzyme B mRNA levels showed the same trend for both exercise programs: they increased slightly directly after exercise and then dropped to levels lower than starting point within 1 h and 24 h after exercise. The latter trend was more noticeable for CONT exercise. Telomeres were studied by quantification of telomere length, telomere ratio and p16 expression (Additional file 5B). None of these three parameters changed significantly in terms of both exercise programs and sample time, even though the telomere ratio tended to be lower 24 h after HIT and CONT exercise.

### HIT had the biggest impact on the number of functionally active VSTs

**Fig. 2:**
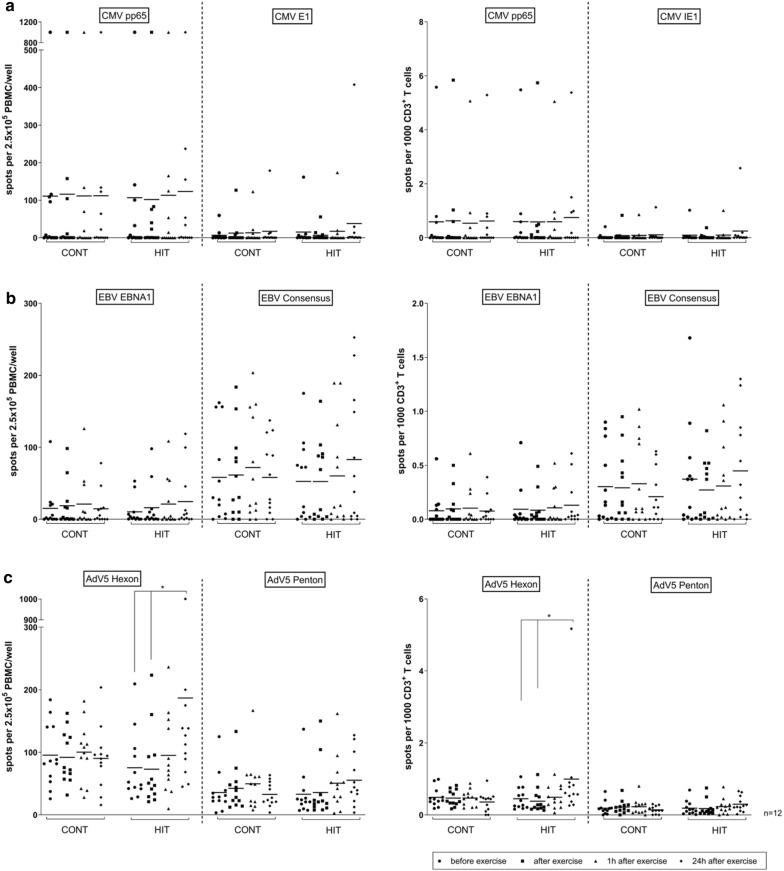
Impact of a single continuous and interval exercise on antigen-specific T-cell responses. Peripheral blood samples of healthy donors (n = 12) were analysed at different time points before and after a single 30 min continuous (CONT) or interval (HIT) exercise (before, directly after, 1 h after and 24 h after exercise). Isolated PBMCs were stimulated overnight with **a** CMV-, **b** EBV- and **c** AdV-specific peptide pools (CMV pp65, CMV IE1, EBV EBNA1, EBV Consensus, AdV5 Hexon and AdV5 Penton) and frequencies of functional-active virus-specific T cells were determined by IFN-γ EliSpot assay. Negative and positive controls were carried out by using either medium without stimuli or 1 μg/ml staphylococcal enterotoxins B (SEB). Results are indicated as the number of spots per 2.5 × 10^5^ cells/well (spw) and spots per 1000 CD3^+^ T cells, respectively after substracting the number of spw of the respective negative control. Positive controls showed a positive result for all analysed samples detected as TNTC (too numerous to count, TNTC ≥ 1000 spw). Results are displayed as mean ± SD. Asterisks indicate statistically significant differences between levels of induced cytokine responses (*p < 0.05)

The exercise-induced change in the number of functionally active VSTs against the investigated peptide pools derived from CMV, EBV and AdV was measured by IFN-γ EliSpot assay at different time points before and after HIT and CONT (Fig. 2). The positive control showed a positive result for all analysed samples specified as TNTC (too numerous to count, TNTC ≥ 1000 spw). Overall, donors exhibited a higher increase 24 h after HIT than 24 h after CONT exercise, which induced almost no or minimal change. The change in the number of functionally active VSTs, expressed as the number of spots per 1000 CD3^+^ T cells from before to 24 h after exercise, was 1.2-fold CMV pp65, 2.6-fold CMV IE-1 (Fig. 2a), 1.3-fold EBV EBNA1, 1.2-fold EBV Consensus (Fig. 2b), 1.6-fold AdV5 Hexon, 2.2-fold AdV5 Penton (Fig. 2c) for HIT compared to 1.0-fold CMV pp65, 2.7-fold CMV IE-1, 0.7-fold EBV EBNA1, 0.7-fold EBV Consensus 0.7-fold, 0.8-fold AdV5 Hexon and AdV5 Penton for CONT. Even though this trend was strong, it only reached statistical significance with AdV5 Hexon directly after HIT exercise, and a 1.6-fold increase occurred within 24 h (p < 0.05, Fig. 2c).

Comparing the effect of HIT and CONT with the two-sample t approach adjusting for period, there was no significant difference between the programs except for AdV5 Penton and EBV Consensus 24 h after exercise (p = 0.013 and p = 0.006), where HIT had the bigger effect (Additional file 6).

**Fig. 3:**
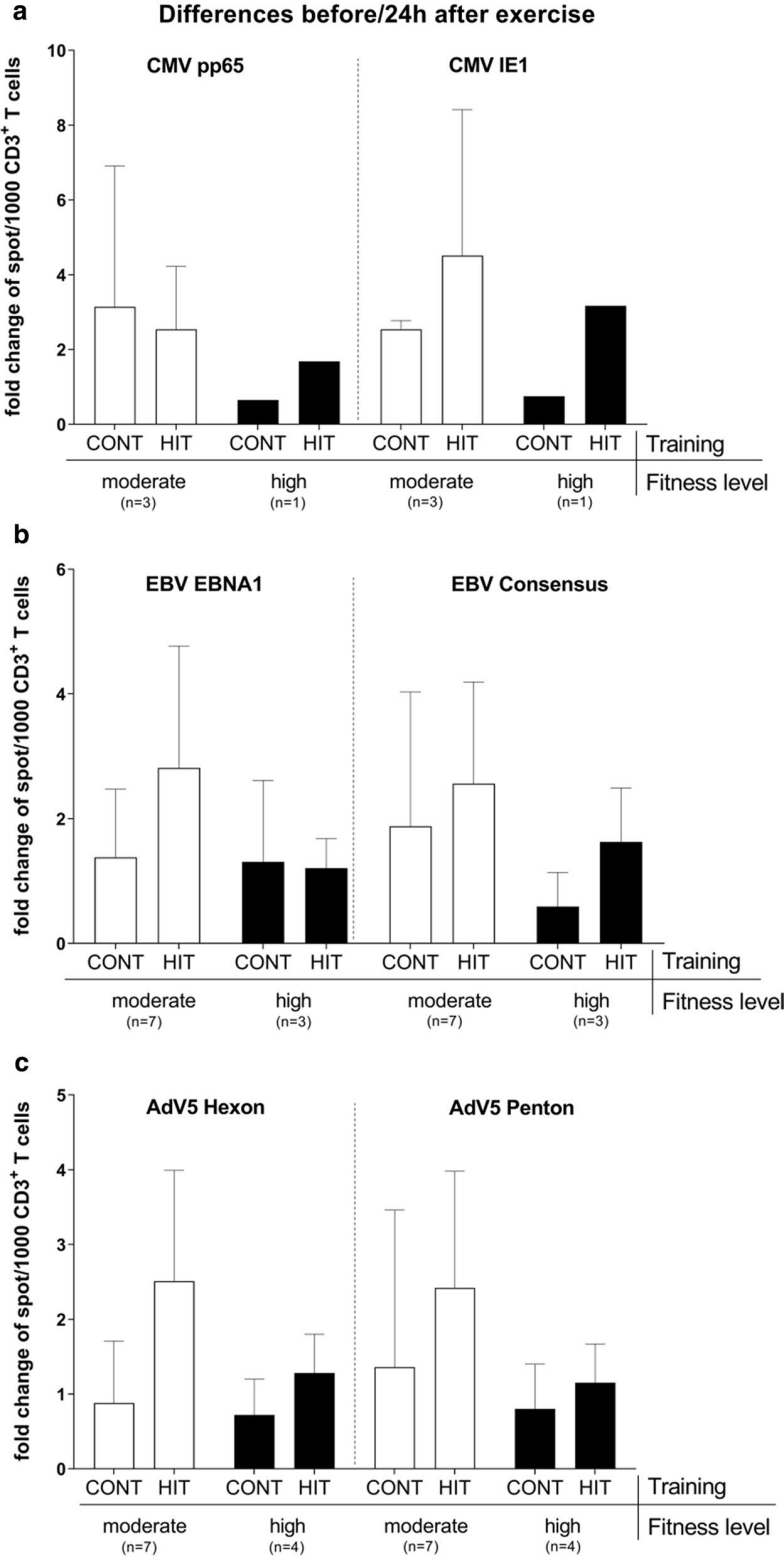
Changes in frequencies of VSTs between moderately and highly fit donors. Donors were additionally classified into a moderately and a highly fit group according to the International Physical Activity Questionnaire (IPAQ). Frequencies of functional-active virus-specific T cells were determined by IFN-γ EliSpot assay. Negative and positive controls were carried out by using either medium or 1 μg/ml staphylococcal enterotoxins B (SEB). Results **a** in response to CMV, **b** EBV and **c** AdV from before and 24 h after exercise are shown as fold changes of spots per well per 1000 CD3^+^ T cells after subtracting those of the respective negative control. Positive control showed a positive result for all analysed samples detected as TNTC (too numerous to count, TNTC ≥ 1000 spw). Results are displayed as mean ± SD

Concerning the effects of the individual donor fitness level on VST activation (Fig. 3), HIT most effectively boosted VSTs against AdV- and EBV-derived antigens in moderately fit donors (AdV: up to 2.5-fold increased; EBV: up to 2.8-fold increased) and was less efficient in augmenting the immunodominant CMV-derived antigen pp65 (2.5-fold increased) than the less immunodominant CMV IE1 (4.5-fold increased).

### Proof-of concept: enrichment of antiviral T cells using small scale Cytokine Secretion Assay

**Fig. 4:**
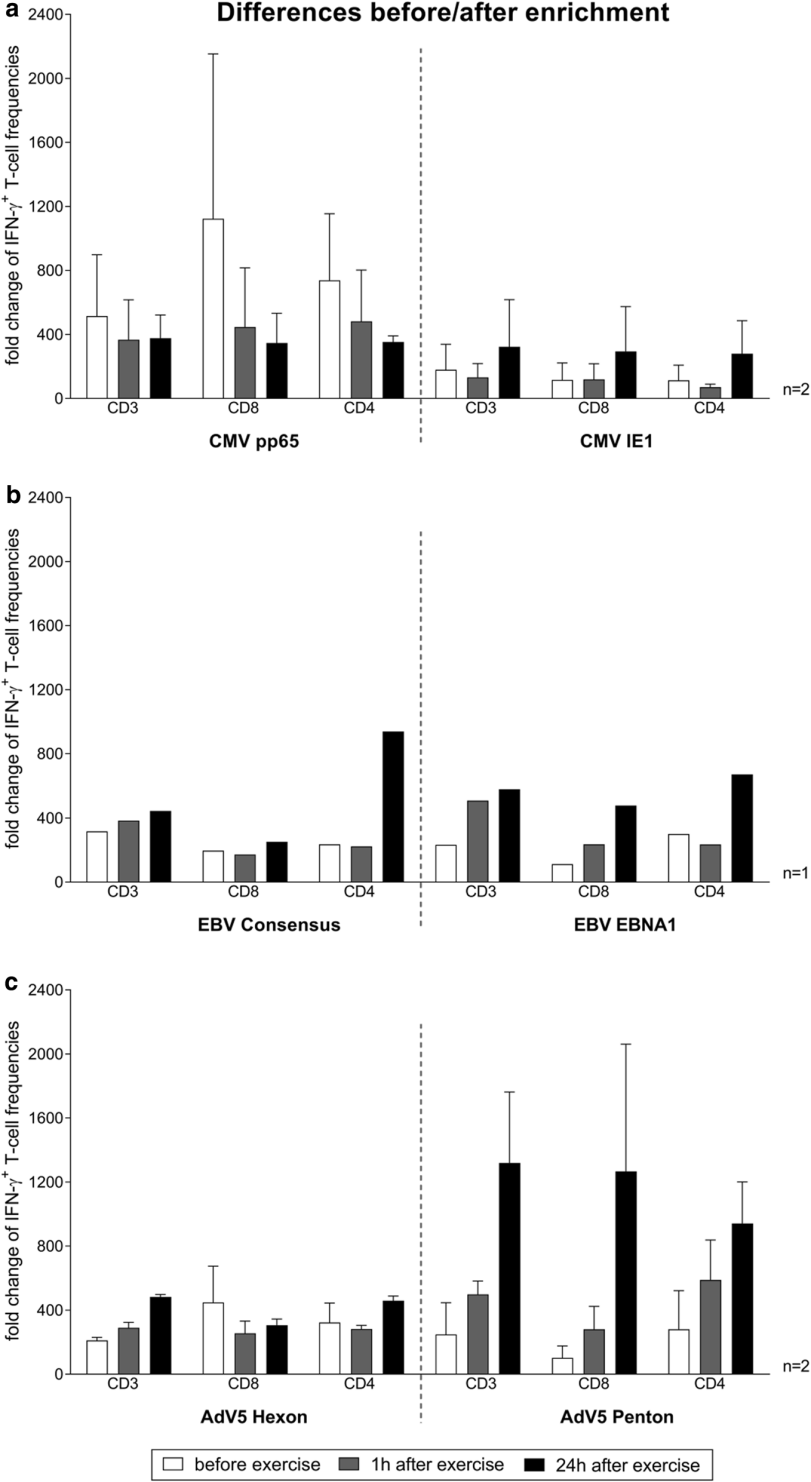
Effects. of a single continuous and interval exercise on the activation capacity of functional IFN-γ^+^ antigen-specific T cells. Peripheral blood samples of healthy donors (n = 6) were analysed before, 1 h after and 24 h after a single 30 min interval (HIT) using IFN-γ Cytokine Secretion Assay (CSA). Isolated PBMCs were stimulated overnight with **a** CMV-, **b** EBV- and **c** AdV-specific peptide pools (CMV pp65, CMV IE1, EBV EBNA1, EBV Consensus, AdV5 Hexon and AdV5 Penton). Negative control was carried out by using unstimulated PBMCs. The relevant cell fractions before and after enrichment were used for a detailed flow cytometric analysis of IFN-γ^+^ T-cell subsets. Results are shown as fold changes of the frequency of CD3^+^/IFN-γ^+^, CD8^+^/IFN-γ^+^ and CD4^+^/IFN-γ^+^ cells after subtracting those of the respective negative control. CMV and AdV results are displayed as mean ± SD

Encouraged by the positive effect of HIT on VST activation identified by the IFN-γ EliSpot assay, we also examined its effect on IFN-γ-secreting T cells using the IFN-γ CSA, which revealed a similar outcome (Fig. 4). All antigens except the highly immunodominant antigen CMV pp65 [46] evoked fold increases in CD3^+^ T cells after short stimulation with the largest increased detected 24 h after exercise. The fold increases at 24 h after exercise compared to baseline were as follows: CD3^+^/IFN-γ^+^: 0.73-fold CMV pp65, 1.8-fold CMV IE-1, 2.5-fold EBV EBNA1, 1.4-fold EBV Consensus, 2.3-fold AdV5 Hexon and 5.3-fold AdV5 Penton. Overall, AdV5 Penton exhibited the greatest increase (CD3^+^/IFN-γ^+^: 5.3-fold increase 24 h after exercise, CD8^+^/IFN-γ^+^: 12.4-fold increase 24 h after exercise, CD4^+^/IFN-γ^+^: 3.4-fold increase 24 h after exercise).

### A single bout of exercise directly affected CD8^+^ T-cell cholesterol and activation levels

**Fig. 5:**
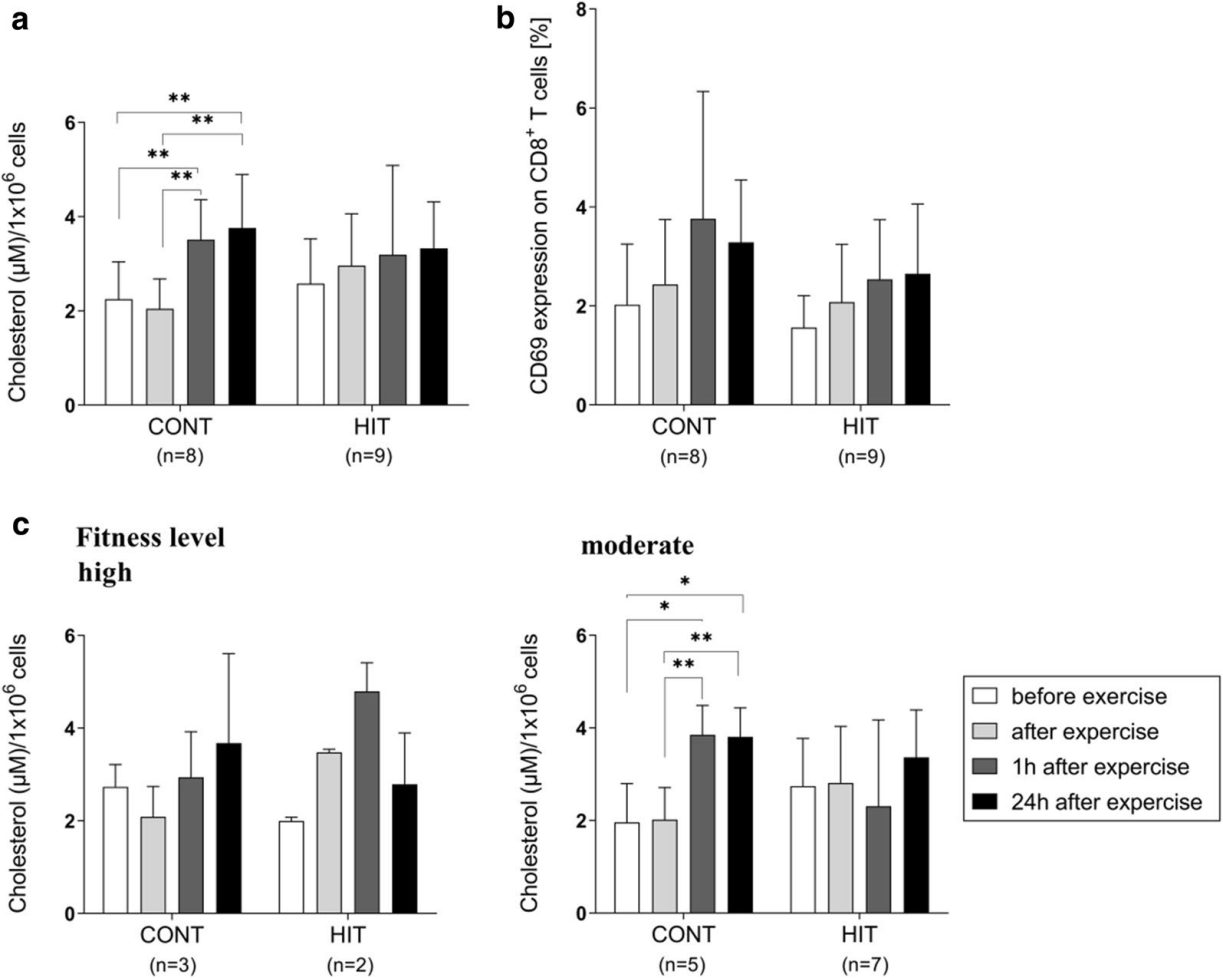
Effects of a single continuous and interval exercise on CD8^+^ T-cell cholesterol and activation levels. Peripheral blood samples of healthy donors (n = 12) were analysed at different time points before and after exercise (before, directly after, 1 h after and 24 h after exercise), in which each donor at least completed one training program (CONT, 8/12 donors and HIT, 9/12 donors) to quantify the level of cholesterol and to analyse the CD69 expression on CD8^+^ T cells. Data are shown in total as mean ± SD. Statistically significant difference is indicated by (*p < 0.05) and (**p < 0.01). Cholesterol and cell activation related parameters were obtained as **a** Cholesterol (µM)/1 × 10^6^ cells (Amplex Red Cholesterol Assay Kit) and **b** CD69 expression on CD8^+^ T cells (flow cytometry). **c** Additionally, the cholesterol level was analysed regarding the donors’ fitness level. 4/12 donors were classified as donors with a high fitness levels (CONT n = 3/4 and HIT n = 2/4) and 8/12 as donors with a moderate fitness level (CONT n = 5/8 and HIT n = 7/8)

A single bout of CONT or HIT resulted in a steady increase in cholesterol in isolated CD8^+^ T cells (Fig. 5). The overall change from before to 24 h after exercise was 1.46-fold (Fig. 5). This change correlated to expression levels of the activation marker CD69 on isolated CD8^+^ T cells; the total change was 1.66-fold before to 24 h after exercise (Fig. 5b). At 24 h post-exercise, the increase in cholesterol was higher after CONT exercise (1.67-fold increase, CD69: 1.66-fold change) than after HIT (1.29-fold change, CD69: 1.69-fold change). Donor-related comparison confirmed these results: individual donors showed higher increases in cholesterol 24 h after CONT (1.92-fold change) than 24 h after HIT (1.09-fold change), which only led to a slight increase in cholesterol (Additional file 7). Regarding the relationship between donor fitness level and cholesterol (Fig. 5c), the highest cholesterol levels after a single bout of HIT exercise were detected in donors with a high fitness level (2.40-fold change 1 h after exercise), whereas those observed after a single bout of CONT exercise occurred in donors with a moderate fitness level (1.96-fold change 1 h after exercise).

## Discussion

Research has shown that physical exercise augments the number of circulating VSTs in peripheral blood [41]. The current study demonstrates for the first time that (1) HIT is the preferable type of exercise for this purpose, (2) the optimal time for efficient augmentation is 24 h prior to leukapheresis, and (3) HIT most effectively boosts the proliferation of VSTs with low resting frequencies in moderately fit donors. These results may have important implications for VST production with regard to coaching donors prior to leukapheresis.

Jamurtas et al. [52] recently observed that WBC and lymphocyte counts increase immediately after both continuous exercise and high-intensity interval training, while monocyte counts remain unchanged. The fact that the increase after HIT was significantly greater suggests that the VST yield may also be augmented post interval training. In agreement with our results, the observed leuko- and lymphocytosis returned to baseline within 24 h after training. Walsh et al. reported that the same biphasic pattern of mobilization can be observed for T cells and is mainly due to the direct and indirect effects of epinephrine [40]. It has been shown that a single bout of various types of continuous exercise mobilizes VSTs specific for latent and non-latent herpesviruses (i.e. CMV, EBV and AdV) to the peripheral bloodstream and has a beneficial effect on their in vitro expansion [25, 41].

Accordingly, we also observed an increase in leukocyte and lymphocyte counts directly after both exercise protocols, while the number and function of CD3^+^ T cells was highest 24 h after exercise. This response could be due to the sympathetically mediated elevation of stress levels directly after exercise resulting in higher glucocorticoid production and, therefore, temporary inhibition of cytokine production until recovery [53].

Strikingly, we observed the highest T-cell response after 30 min of HIT against AdV5 Hexon and AdV5 Penton in both IFN-γ EliSpot Assay and IFN-γ CSA, while CMV pp65 did not respond as much to exercise. HIT appears to augment IFN-γ secretion, especially in the case of circulating memory VSTs with low precursor frequencies, like AdV-specific T cells [46]. Conversely, T cells against the highly immunodominant antigen CMV pp65 [54], which have higher frequencies in the circulation of healthy donors, did not respond as much to exercise. This finding suggests that HIT is most beneficial for boosting antigen-specific T cells with low precursor frequencies.

Interestingly, these beneficial effects on mobilization and function were more distinct in moderately fit individuals. It is known that regular prolonged exercise promotes immunosenescence of T cells [55]. This could be a reason why the highly fit individuals did not respond as much to a single session of exercise. Another reason could be that highly fit individuals are more used to exercise stimuli and therefore have a lower VST activation response to exercise.

Among other co-stimulatory factors, a key component of this activation response is the clustering of T-cell receptors (TCR) at the centre of the T-cell/antigen-presenting-cell interface [56]. Recent studies have demonstrated that enhanced cholesterol levels of the whole cell and the plasma membrane are linked to increased functionality of activated CD8^+^ T cells resulting in enhanced TCR clustering and signalling as well as more efficient formation of the immunological synapse [57–60]. For this reason, we investigated whether a single bout of HIT or CONT results in higher cholesterol levels, which could lead to increased activation and cytotoxic function of CD8^+^ T cells by strengthening TCR signaling. Indeed, cholesterol levels in CD8^+^ T cells increased in response to exercise. Even though the difference between the two exercise protocols was not statistically significant, cholesterol levels and CD69 expression in CD8^+^ T cells increased significantly after continuous exercise, predominantly in moderately fit donors. As early marker for VSTs activation [61], the observed increases in CD69 and cholesterol correlated well with higher IFN-γ secretion. In a comprehensive future study, we will investigate the impact and modulatory role of cholesterol on VST activation and function.

Another interesting trend could be seen with HSP70. Different types of stress, one of which is exercise, induces HSP70 expression in order to adapt organisms appropriately [62, 63]. In a rat model, it was shown that at a high-intensity workload, HSP70 increases while the proliferation response decreases [62]. This corresponds to our findings concerning HSP70 and the proliferation marker Ki67.

However, it is not surprising that a single bout of exercise did not alter measures of replicative senescence such as telomere length. Habitual exercise slows the age-related decrease in telomere length. However, this seems to be one of the long-term effects of regular exercise [64] and is not observed after a single exercise bout.

Although they are now used as second-line treatment in patients who do not respond to first-line treatments such as cidofovir for AdV infections, researchers are evaluating the usefulness of VSTs as an adjuvant to standard agents in the initial treatment [65].

In the present study, the peak VST frequencies differed from the peak levels of general lymphocyte counts: HIT did not evoke the highest VST activity when the CD4^+^ and CD8^+^ frequencies peaked. In fact, a higher antigen response was observed 24 h after HIT even though frequencies of the above-mentioned T-cell subsets were higher at other time points. Spielmann et al. found that although continuous exercise augments the ex vivo manufacture of VSTs, this effect does not appear to be due to numerical changes in the number of VSTs stimulated; hence, they deemed it unlikely that the beneficial effect of exercise achieved in their ex vivo expansion experiments could be replicated by simply drawing larger volumes of blood [39].

Thus, a single bout of HIT exercise 24 h before donation can serve as a simple and economical adjuvant to significantly improve adoptive T-cell therapy with respect to manufacturing functional-active antigen-specific T-cell products in sufficient cell numbers. This is especially effective in the case of memory VSTs with low precursor frequencies.

## Conclusions

A single bout of exercise, preferably HIT 24 h prior to blood donation, can significantly improve T-cell enrichment in VST production for adoptive T-cell immunotherapy. Exercise has benefits, especially for viral antigens for which most healthy individuals possess less frequent T cells, particularly in donors who are moderately fit. As single bouts of exercise in general seem to be beneficial, they can be recommended to all donors.

## Supplementary information

**Additional file 1.** Representative flow cytometric plots. Representative flow cytometry plots illustrating the gating strategy used to analyse (A) the cellular immune status in whole blood sample on a single-cell platform using TruCount tubes, (B) the enrichment efficiency of IFN-γ^+^ VSTs selected from PBMCs using the IFN-γ cytokine secretion assay, and (C) the activation level of isolated CD8^+^ T cells using CD69 as specific marker before and after exercise.

**Additional file 2.** Changes on the cellular immune status after continuous and interval exercise. Peripheral blood samples of healthy donors (n = 12) were analysed at different time points before and directly after a single 30 min continuous (CONT) or interval (HIT) exercise (before, directly after, 1 h after and 24 h after exercise) using a haemocytometer (A–D) or flow cytometry (E–F). (A) White blood cell (WBC), (B) lymphocyte, (C) granulocyte and (D) monocyte counts were detected as absolute counts (×10^3^/µl). Data are shown as normalized cell counts (A–D), normalized frequencies of (E) CD3^+^ T cells and the (F) normalized CD4/CD8 ratio. Data are mean ± SD.

**Additional file 3.** Changes on standard blood counts and the cellular immune status after continuous and interval exercise. Peripheral blood samples of healthy donors (n = 12) were analysed by flow cytometry at different time points before and after a single 30 min continuous (CONT) or interval (HIT) exercise (before, directly after, 1 h after and 24 h after exercise). Determined frequencies of CD45^+^ leukocytes, CD14^+^ monocytes, CD19^+^ B cells, CD3^+^/CD56^+^ NKT cells, and CD3^−^/CD56^+^ NK cells with their bright and dim subsets are displayed as normalized frequencies. Data are mean ± SD.

**Additional file 4.** Changes in standard blood counts after continuous and interval exercise differentiating between moderately and highly fit donors. Peripheral blood samples of healthy donors (n = 12) were analysed using a haemocytometer at different time points before and after a single 30 min continuous (CONT) or interval (HIT) exercise (before, directly after, 1 h after and 24 h after exercise). Donors were grouped according to their fitness level (moderate, n = 8, and high, n = 4, tested with the International Physical Activity Questionnaire, IPAQ). (A) Lymphocyte, granulocyte, and monocyte frequency is shown as percentage of the total leukocyte count. (B) White blood cell (WBC), lymphocyte, granulocyte, and monocyte counts are shown as absolute counts (×10^3^/µl blood). Data are shown as mean ± SD.

**Additional file 5.** Effects of a single continuous and interval exercise on stress, proliferation markers, T-cell cytotoxicity and parameters of cellular age. Peripheral blood samples of healthy donors (n = 12) were analysed at different time points before and after a single 30 min continuous (CONT) or interval (HIT) exercise (before, directly after, 1 h after and 24 h after exercise) using real-time PCR for quantification of (A) HSP70- and Ki67-, IFN-γ-, and granzyme B-mRNA levels. Constitutively expressed GAPDH gene was used as the reference standard for normalization of mRNA levels. RQ values were calculated by the delta–delta CT method. (B) Blood samples were further analysed regarding p16 levels, telomere ratio and telomere base pairs. Results are displayed as RQ, with the value from before exercise being the base line, and shown as mean ± SD.

**Additional file 6.** Impact of a single continuous and interval exercise on antigen-specific T-cell responses. Peripheral blood samples of healthy donors (n = 12) were analysed separately with the two-sample t approach adjusting for period at different time points before and after a single 30 min continuous (CONT) or interval (HIT) exercise (before, directly after, 1 h after and 24 h after exercise). Isolated PBMCs were stimulated overnight with CMV-, EBV- and AdV-specific peptide pools (CMV pp65, CMV IE1, EBV EBNA1, and EBV Consensus, AdV5 Hexon, and AdV5 Penton) and frequencies of functional-active virus-specific T cells were determined by IFN-γ EliSpot assay as spots per 1000 CD3^+^ T cells

**Additional file 7.** Effects of a single continuous and interval exercise on CD8^+^ T-cell cholesterol and activation levels in a donor-related setting. Peripheral blood samples of healthy donors (n = 5) were analysed at different time points before and after a single 30 min continuous (CONT) or interval (HIT) exercise (before, directly after, 1 h after and 24 h after exercise) to (A) quantify the level of cholesterol (µM)/1 × 10^6^ cells (Amplex Red Cholesterol Assay Kit) and (B) to obtain the expression of CD69 on CD8^+^ T cells (flow cytometry). Data are shown in a donor-related setting as mean ± SD. Statistically significant difference is indicated by (*p < 0.05) and (**p < 0.01).

## Data Availability

All data generated or analysed during this study are included in this published article and its additional files. The datasets used and analysed during the current study are available from the corresponding author on reasonable request.

## Abbreviations

AdV: Adenovirus
APC/Cy7: Anti-CD45-allophycocyanin plus cyanin-7
CBC: Complete blood counts
CM: Culture medium
CMV: Cytomegalovirus
CONT: Continuous exercise
CSA: Cytokine Secretion Assay
DLI: Donor lymphocyte infusions
EBV: Epstein–Barr-virus
EliSpot assay: Enzyme-linked immunospot assay
FITC: Anti-CD3-fluorescein isothiocyanate
GAPDH: Glyceraldehyde 3-phosphate dehydrogenase
GvHD: Graft-versus-host disease
GXT: Graded exercise test
HHV6: Human herpesvirus 6
HIHVT: High Intensity High Volume Training
HIT: High Intensity Interval Training
HR: Heart rate
HSCT: Hematopoietic stem cell transplantation
HSP70: Heat shock protein 70
IFN-γ: Interferon-γ
IPAQ: International Physical Activity Questionnaire
mAbs: Monoclonal antibodies
MHH: Hannover Medical School
NK cells: Natural killer cells
PBMCs: Peripheral blood mononuclear cells
PE: Phycoerthrin
PerCP: Peridinin chlorophyll protein
P_max_: Maximum power output
pMHC: Peptide major histocompatibility complex
PTLD: Post-transplant lymphoproliferative disorder
qPCR: Quantitative real-time PCR
SEB: Staphylococcal enterotoxin B
spw: Spots per well
SOT: Solid organ transplantation
TCR: T-cell receptors
VST: Virus-specific T cells
WBC: White blood cells
7AAD: 7-Amino-actinomycin D

## References

[1] Holmes-Liew CL, Holmes M, Beagley L, Hopkins P, Chambers D, Smith C, Khanna R (2015). Adoptive T-cell immunotherapy for ganciclovir-resistant CMV disease after lung transplantation. Clin Transl Immunol.

[2] Qian C, Campidelli A, Wang Y, Cai H, Venard V, Jeulin H, Dalle JH, Pochon C, D’Aveni M, Bruno B (2017). Curative or pre-emptive adenovirus-specific T cell transfer from matched unrelated or third party haploidentical donors after HSCT, including UCB transplantations: a successful phase I/II multicenter clinical trial. J Hematol Oncol.

[3] Tomblyn M, Chiller T, Einsele H, Gress R, Sepkowitz K, Storek J, Wingard JR, Young JA, Boeckh MJ, Center for International B (2009). Guidelines for preventing infectious complications among hematopoietic cell transplantation recipients: a global perspective. Biol Blood Marrow Transplant.

[4] Gratwohl A, Brand R, Frassoni F, Rocha V, Niederwieser D, Reusser P, Einsele H, Cordonnier C, Acute, Chronic Leukemia Working P (2005). Cause of death after allogeneic haematopoietic stem cell transplantation (HSCT) in early leukaemias: an EBMT analysis of lethal infectious complications and changes over calendar time. Bone Marrow Transplant.

[5] Kaeuferle T, Krauss R, Blaeschke F, Willier S, Feuchtinger T (2019). Strategies of adoptive T-cell transfer to treat refractory viral infections post allogeneic stem cell transplantation. J Hematol Oncol.

[6] Symeonidis N, Jakubowski A, Pierre-Louis S, Jaffe D, Pamer E, Sepkowitz K, O’Reilly RJ, Papanicolaou GA (2007). Invasive adenoviral infections in T-cell-depleted allogeneic hematopoietic stem cell transplantation: high mortality in the era of cidofovir. Transpl Infect Dis.

[7] Marinho-Dias J, Baldaque I, Pinho-Vaz C, Leite L, Branca R, Campilho F, Campos A, Medeiros R, Sousa H (2019). Association of EpsteinBarr virus infection with allogeneic hematopoietic stem cell transplantation in patients in Portugal. Mol Med Rep.

[8] Marques HH, Shikanai-Yasuda MA, dAzevedo LS, Caiaffa-Filho HH, Pierrotti LC, Aquino MZ, Lopes MH, Maluf NZ, Campos SV, Costa SF (2014). Management of post-transplant Epstein–Barr virus-related lymphoproliferative disease in solid organ and hematopoietic stem cell recipients. Rev Soc Bras Med Trop.

[9] Diaz L, Rosales J, Rosso F, Rosales M, Estacio M, Manzi E, Jaramillo FJ (2019). Cytomegalovirus disease in patients with hematopoietic stem cell transplantation, experience over 8 years. Hematol Transfus Cell Ther.

[10] Liu J, Kong J, Chang YJ, Chen H, Chen YH, Han W, Wang Y, Yan CH, Wang JZ, Wang FR (2015). Patients with refractory cytomegalovirus (CMV) infection following allogeneic haematopoietic stem cell transplantation are at high risk for CMV disease and non-relapse mortality. Clin Microbiol Infect.

[11] Fishman JA (2017). Infection in organ transplantation. Am J Transplant.

[12] Ramanan P, Razonable RR (2013). Cytomegalovirus infections in solid organ transplantation: a review. Infect Chemother.

[13] Choquet S, Varnous S, Deback C, Golmard JL, Leblond V (2014). Adapted treatment of Epstein–Barr virus infection to prevent posttransplant lymphoproliferative disorder after heart transplantation. Am J Transplant.

[14] Frietsch JJ, Michel D, Stamminger T, Hunstig F, Birndt S, Schnetzke U, Scholl S, Hochhaus A, Hilgendorf I (2019). In vivo emergence of UL56 C325Y cytomegalovirus resistance to letermovir in a patient with acute myeloid leukemia after hematopoietic cell transplantation. Mediterr J Hematol Infect Dis.

[15] Bollard CM (2013). Improving T-cell therapy for epstein-barr virus lymphoproliferative disorders. J Clin Oncol.

[16] Sanz J, Andreu R (2014). Epstein–Barr virus-associated posttransplant lymphoproliferative disorder after allogeneic stem cell transplantation. Curr Opin Oncol.

[17] Hiwarkar P, Amrolia P, Sivaprakasam P, Lum SH, Doss H, O’Rafferty C, Petterson T, Patrick K, Silva J, Slatter M (2017). Brincidofovir is highly efficacious in controlling adenoviremia in pediatric recipients of hematopoietic cell transplant. Blood.

[18] Feuchtinger T, Matthes-Martin S, Richard C, Lion T, Fuhrer M, Hamprecht K, Handgretinger R, Peters C, Schuster FR, Beck R (2006). Safe adoptive transfer of virus-specific T-cell immunity for the treatment of systemic adenovirus infection after allogeneic stem cell transplantation. Br J Haematol.

[19] Bollard CM, Kuehnle I, Leen A, Rooney CM, Heslop HE (2004). Adoptive immunotherapy for posttransplantation viral infections. Biol Blood Marrow Transplant.

[20] Abraham AA, John TD, Keller MD, Cruz CRN, Salem B, Roesch L, Liu H, Hoq F, Grilley BJ, Gee AP (2019). Safety and feasibility of virus-specific T cells derived from umbilical cord blood in cord blood transplant recipients. Blood Adv.

[21] Arasaratnam RJ, Leen AM (2015). Adoptive T cell therapy for the treatment of viral infections. Ann Transl Med.

[22] Lindemann M, Eiz-Vesper B, Steckel NK, Tischer S, Fiedler M, Heinold A, Klisanin V, Maecker-Kolhoff B, Blasczyk R, Horn PA (2018). Adoptive transfer of cellular immunity against cytomegalovirus by virus-specific lymphocytes from a third-party family donor. Bone Marrow Transplant..

[23] Maecker-Kolhoff B, Eiz-Vesper B (2015). Broad spectrum antiviral T cells for viral complications after hematopoietic stem cell transplantation. Ann Transl Med.

[24] Qian C, Wang Y, Reppel L, D’Aveni M, Campidelli A, Decot V, Bensoussan D (2018). Viral-specific T-cell transfer from HSCT donor for the treatment of viral infections or diseases after HSCT. Bone Marrow Transplant.

[25] Schmitt A, Tonn T, Busch DH, Grigoleit GU, Einsele H, Odendahl M, Germeroth L, Ringhoffer M, Ringhoffer S, Wiesneth M (2011). Adoptive transfer and selective reconstitution of streptamer-selected cytomegalovirus-specific CD8+ T cells leads to virus clearance in patients after allogeneic peripheral blood stem cell transplantation. Transfusion.

[26] Schultze-Florey RE, Tischer-Zimmermann S, Heuft HG, Priesner C, Lamottke B, Heim A, Sauer M, Sykora KW, Blasczyk R, Eiz-Vesper B, Maecker-Kolhoff B (2020). Transfer of hexon- and penton-selected adenovirus-specific T cells for refractory adenovirus infection after haploidentical stem cell transplantation. Transpl Infect Dis..

[27] Schultze-Florey RE, Tischer S, Kuhlmann L, Hundsdoerfer P, Koch A, Anagnostopoulos I, Ravens S, Goudeva L, Schultze-Florey C, Koenecke C (2018). Dissecting Epstein–Barr virus-specific T-cell responses after allogeneic EBV-specific T-cell transfer for central nervous system posttransplant lymphoproliferative disease. Front Immunol.

[28] Priesner C, Esser R, Tischer S, Marburger M, Aleksandrova K, Maecker-Kolhoff B, Heuft HG, Goudeva L, Blasczyk R, Arseniev L (2016). Comparative analysis of clinical-scale IFN-gamma-positive T-cell enrichment using partially and fully integrated platforms. Front Immunol.

[29] Tischer S, Priesner C, Heuft HG, Goudeva L, Mende W, Barthold M, Kloess S, Arseniev L, Aleksandrova K, Maecker-Kolhoff B (2014). Rapid generation of clinical-grade antiviral T cells: selection of suitable T-cell donors and GMP-compliant manufacturing of antiviral T cells. J Transl Med.

[30] Feucht J, Joachim L, Lang P, Feuchtinger T (2013). Adoptive T-cell transfer for refractory viral infections with cytomegalovirus, Epstein–Barr virus or adenovirus after allogeneic stem cell transplantation. Klin Padiatr.

[31] Geyeregger R, Freimuller C, Stemberger J, Artwohl M, Witt V, Lion T, Fischer G, Lawitschka A, Ritter J, Hummel M (2014). First-in-man clinical results with good manufacturing practice (GMP)-compliant polypeptide-expanded adenovirus-specific T cells after haploidentical hematopoietic stem cell transplantation. J Immunother.

[32] Kallay K, Kassa C, Reti M, Karaszi E, Sinko J, Goda V, Strehn A, Csordas K, Horvath O, Szederjesi A (2018). Early experience With CliniMACS Prodigy CCS (IFN-gamma) system in selection of virus-specific T cells from third-party donors for pediatric patients with severe viral infections after hematopoietic stem cell transplantation. J Immunother.

[33] Uhlin M, Gertow J, Uzunel M, Okas M, Berglund S, Watz E, Brune M, Ljungman P, Maeurer M, Mattsson J (2012). Rapid salvage treatment with virus-specific T cells for therapy-resistant disease. Clin Infect Dis.

[34] Withers B, Blyth E, Clancy LE, Yong A, Fraser C, Burgess J, Simms R, Brown R, Kliman D, Dubosq MC (2017). Long-term control of recurrent or refractory viral infections after allogeneic HSCT with third-party virus-specific T cells. Blood Adv.

[35] Withers B, Clancy L, Burgess J, Simms R, Brown R, Micklethwaite K, Blyth E, Gottlieb D (2018). Establishment and operation of a third-party virus-specific T cell bank within an allogeneic stem cell transplant program. Biol Blood Marrow Transplant.

[36] Bollard CM, Heslop HE (2016). T cells for viral infections after allogeneic hematopoietic stem cell transplant. Blood.

[37] Simpson RJ, Kunz H, Agha N, Graff R (2015). Exercise and the regulation of immune functions. Prog Mol Biol Transl Sci.

[38] Belviranli M, Okudan N, Kabak B. The effects of acute high-intensity interval training on hematological parameters in sedentary subjects. Med Sci. 2017;5. 10.3390/medsci5030015 PMC5635806 29099031

[39] Spielmann G, Bollard CM, Kunz H, Hanley PJ, Simpson RJ (2016). A single exercise bout enhances the manufacture of viral-specific T-cells from healthy donors: implications for allogeneic adoptive transfer immunotherapy. Sci Rep.

[40] Walsh NP, Gleeson M, Shephard RJ, Gleeson M, Woods JA, Bishop NC, Fleshner M, Green C, Pedersen BK, Hoffman-Goetz L (2011). Position statement. Part one: immune function and exercise. Exerc Immunol Rev.

[41] Kunz HE, Spielmann G, Agha NH, O’Connor DP, Bollard CM, Simpson RJ (2018). A single exercise bout augments adenovirus-specific T-cell mobilization and function. Physiol Behav.

[42] Gibala MJ, Little JP, Macdonald MJ, Hawley JA (2012). Physiological adaptations to low-volume, high-intensity interval training in health and disease. J Physiol.

[43] Tjonna AE, Stolen TO, Bye A, Volden M, Slordahl SA, Odegard R, Skogvoll E, Wisloff U (2009). Aerobic interval training reduces cardiovascular risk factors more than a multitreatment approach in overweight adolescents. Clin Sci.

[44] Craig CL, Marshall AL, Sjostrom M, Bauman AE, Booth ML, Ainsworth BE, Pratt M, Ekelund U, Yngve A, Sallis JF, Oja P (2003). International physical activity questionnaire: 12-country reliability and validity. Med Sci Sports Exerc.

[45] Bieling M, Tischer S, Kalinke U, Blasczyk R, Buus S, Maecker-Kolhoff B, Eiz-Vesper B (2018). Personalized adoptive immunotherapy for patients with EBV-associated tumors and complications: evaluation of novel naturally processed and presented EBV-derived T-cell epitopes. Oncotarget..

[46] Sukdolak C, Tischer S, Dieks D, Figueiredo C, Goudeva L, Heuft HG, Verboom M, Immenschuh S, Heim A, Borchers S (2013). CMV-, EBV- and ADV-specific T cell immunity: screening and monitoring of potential third-party donors to improve post-transplantation outcome. Biol Blood Marrow Transplant.

[47] Tischer S, Dieks D, Sukdolak C, Bunse C, Figueiredo C, Immenschuh S, Borchers S, Stripecke R, Maecker-Kolhoff B, Blasczyk R, Eiz-Vesper B (2014). Evaluation of suitable target antigens and immunoassays for high-accuracy immune monitoring of cytomegalovirus and Epstein–Barr virus-specific T cells as targets of interest in immunotherapeutic approaches. J Immunol Methods.

[48] Bunse CE, Fortmeier V, Tischer S, Zilian E, Figueiredo C, Witte T, Blasczyk R, Immenschuh S, Eiz-Vesper B (2015). Modulation of heme oxygenase-1 by metalloporphyrins increases anti-viral T cell responses. Clin Exp Immunol.

[49] Cawthon RM, Smith KR, O’Brien E, Sivatchenko A, Kerber RA (2003). Association between telomere length in blood and mortality in people aged 60 years or older. Lancet.

[50] Melk A, Tegtbur U, Hilfiker-Kleiner D, Eberhard J, Saretzki G, Eulert C, Kerling A, Nelius AK, Homme M, Strunk D (2014). Improvement of biological age by physical activity. Int J Cardiol.

[51] Senn S (2002). Cross-over trials in clinical trials.

[52] Jamurtas AZ, Fatouros IG, Deli CK, Georgakouli K, Poulios A, Draganidis D, Papanikolaou K, Tsimeas P, Chatzinikolaou A, Avloniti A (2018). The effects of acute low-volume HIIT and aerobic exercise on leukocyte count and redox status. J Sports Sci Med.

[53] Gleeson M, Bishop NC (2005). The T cell and NK cell immune response to exercise. Ann Transplant.

[54] Malik A, Adland E, Laker L, Kloverpris H, Fardoos R, Roider J, Severinsen MC, Chen F, Riddell L, Edwards A (2017). Immunodominant cytomegalovirus-specific CD8+ T-cell responses in sub-Saharan African populations. PLoS ONE.

[55] Turner JE, Brum PC (2017). Does regular exercise counter T cell immunosenescence reducing the risk of developing cancer and promoting successful treatment of malignancies?. Oxid Med Cell Longev.

[56] Purtic B, Pitcher LA, van Oers NS, Wulfing C (2005). T cell receptor (TCR) clustering in the immunological synapse integrates TCR and costimulatory signaling in selected T cells. Proc Natl Acad Sci USA.

[57] Yang W, Bai Y, Xiong Y, Zhang J, Chen S, Zheng X, Meng X, Li L, Wang J, Xu C (2016). Potentiating the antitumour response of CD8(+) T cells by modulating cholesterol metabolism. Nature.

[58] Bietz A, Zhu H, Xue M, Xu C (2017). Cholesterol metabolism in T cells. Front Immunol.

[59] Swamy M, Beck-Garcia K, Beck-Garcia E, Hartl FA, Morath A, Yousefi OS, Dopfer EP, Molnar E, Schulze AK, Blanco R (2016). A cholesterol-based allostery model of T cell receptor phosphorylation. Immunity.

[60] Yin Z, Bai L, Li W, Zeng T, Tian H, Cui J (2019). Targeting T cell metabolism in the tumor microenvironment: an anti-cancer therapeutic strategy. J Exp Clin Cancer Res.

[61] Holbrook AK, Peterson HD, Bianchi SA, Macdonald BW, Bredahl EC, Belshan M, Siedlik JA (2019). CD4(+) T cell activation and associated susceptibility to HIV-1 infection in vitro increased following acute resistance exercise in human subjects. Physiol Rep.

[62] Heck TG, Scomazzon SP, Nunes PR, Scholer CM, da Silva GS, Bittencourt A, Faccioni-Heuser MC, Krause M, Bazotte RB, Curi R, Homem de Bittencourt PI (2017). Acute exercise boosts cell proliferation and the heat shock response in lymphocytes: correlation with cytokine production and extracellular-to-intracellular HSP70 ratio. Cell Stress Chaperones.

[63] Hooper PL, Balogh G, Rivas E, Kavanagh K, Vigh L (2014). The importance of the cellular stress response in the pathogenesis and treatment of type 2 diabetes. Cell Stress Chaperones.

[64] LaRocca TJ, Seals DR, Pierce GL (2010). Leukocyte telomere length is preserved with aging in endurance exercise-trained adults and related to maximal aerobic capacity. Mech Ageing Dev.

[65] O’Reilly RJ, Prockop S, Hasan AN, Koehne G, Doubrovina E (2016). Virus-specific T-cell banks for ‘off the shelf‘ adoptive therapy of refractory infections. Bone Marrow Transplant.

