# Supplementary figures and images for: High-intensity interval training in allogeneic adoptive T-cell immunotherapy – a big HIT?
Source: J Transl Med. 2020 Apr 1;18:148. doi: 10.1186/s12967-020-02301-3 (PMC7114817; doi:10.1186/s12967-020-02301-3)

# A

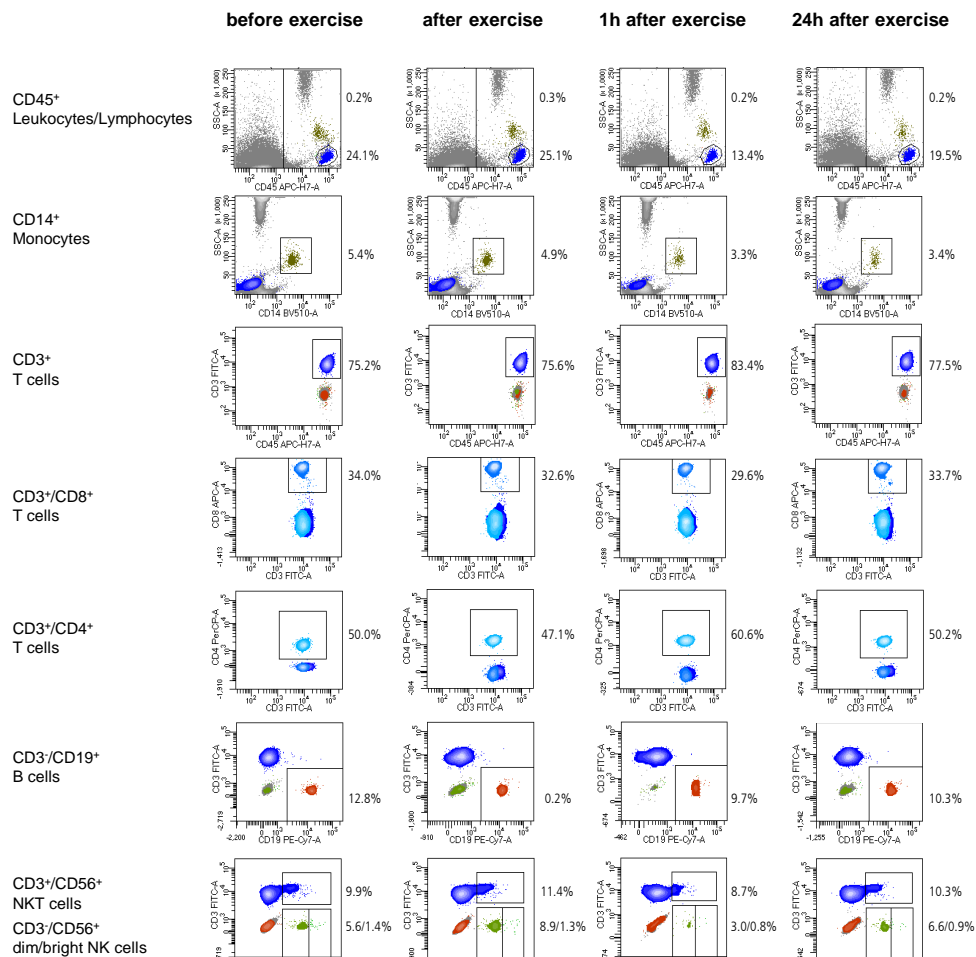

# B

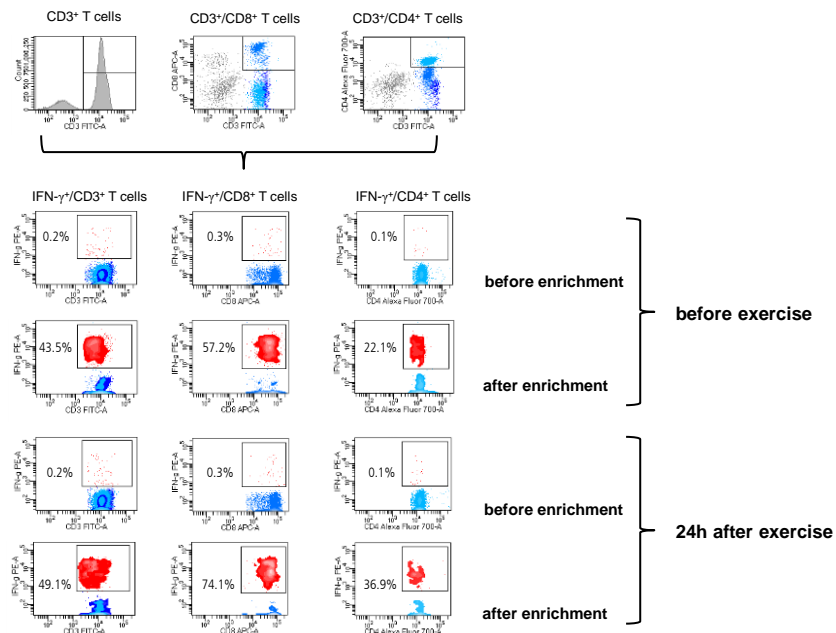

# C

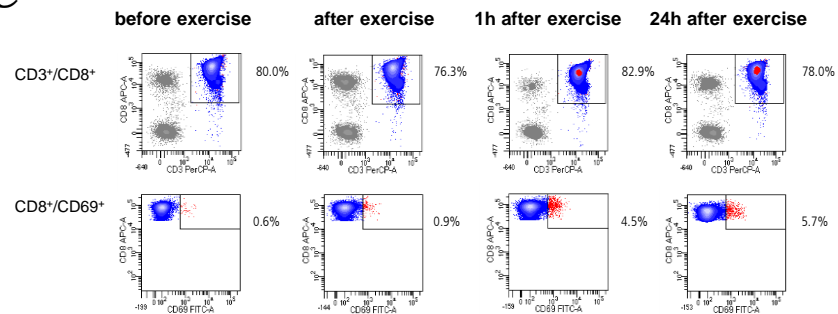

Supplement: Supplementary file 1 — Additional file 1. Representative flow cytometric plots. Representative flow cytometry plots illustrating the gating strategy used to analyse (A) the cellular immune status in whole blood sample on a single-cell platform using TruCount tubes, (B) the enrichment efficiency of IFN-γ+ VSTs selected from PBMCs using the IFN-γ cytokine secretion assay, and (C) the activation level of isolated CD8+ T cells using CD69 as specific marker before and after exercise. [file 12967_2020_2301_MOESM1_ESM.pdf]

**A**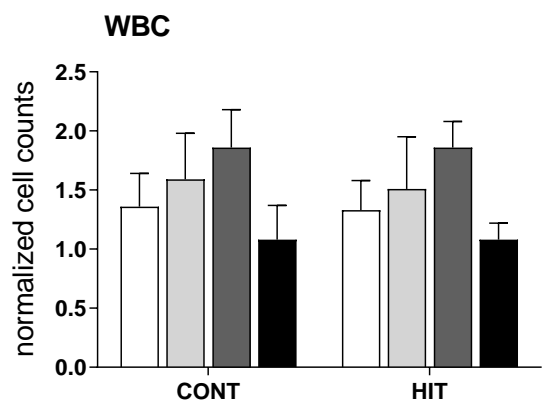**B**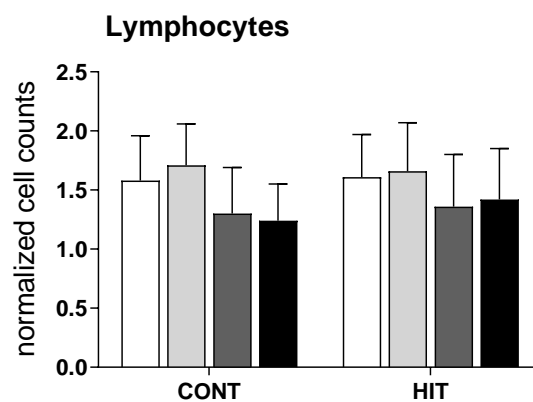**C**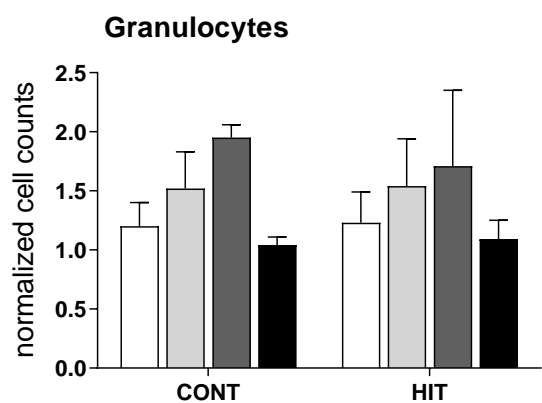**D**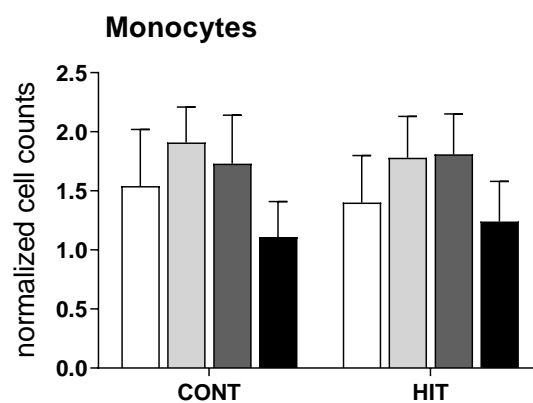**E**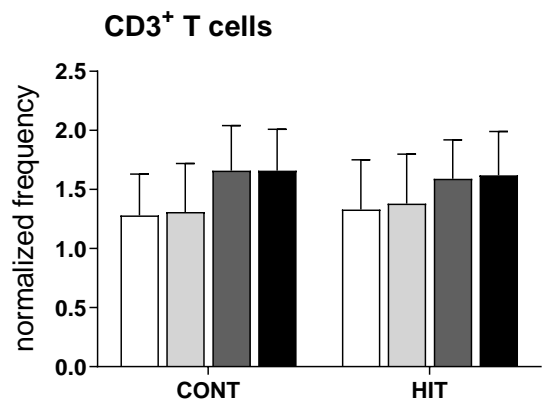**F**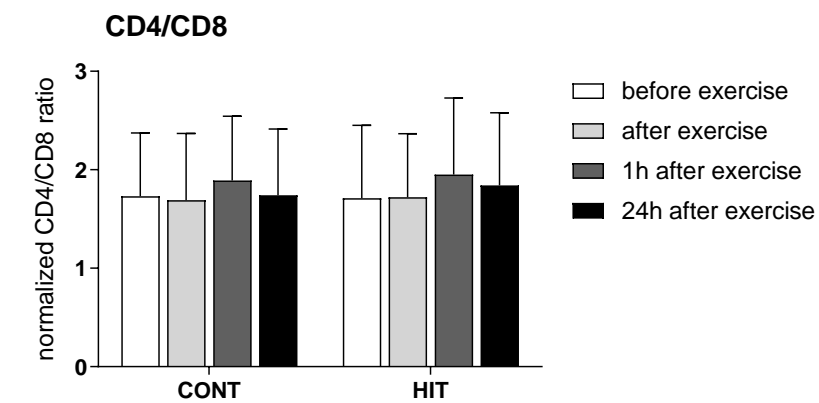

Supplement: Supplementary file 2 — Additional file 2. Changes on the cellular immune status after continuous and interval exercise. Peripheral blood samples of healthy donors (n = 12) were analysed at different time points before and directly after a single 30 min continuous (CONT) or interval (HIT) exercise (before, directly after, 1 h after and 24 h after exercise) using a haemocytometer (A–D) or flow cytometry (E–F). (A) White blood cell (WBC), (B) lymphocyte, (C) granulocyte and (D) monocyte counts were detected as absolute counts (×103/µl). Data are shown as normalized cell counts (A–D), normalized frequencies of (E) CD3+ T cells and the (F) normalized CD4/CD8 ratio. Data are mean ± SD. [file 12967_2020_2301_MOESM2_ESM.pdf]

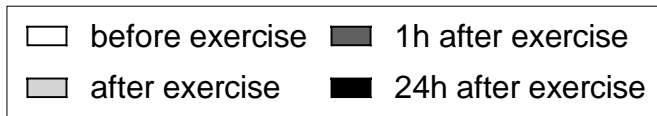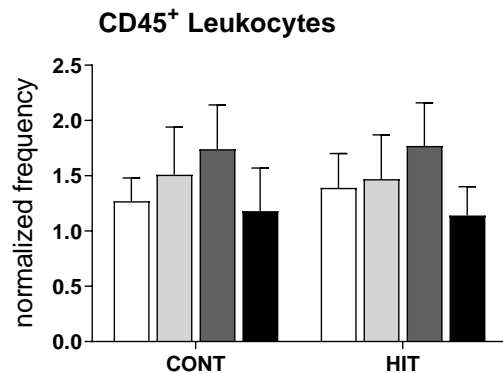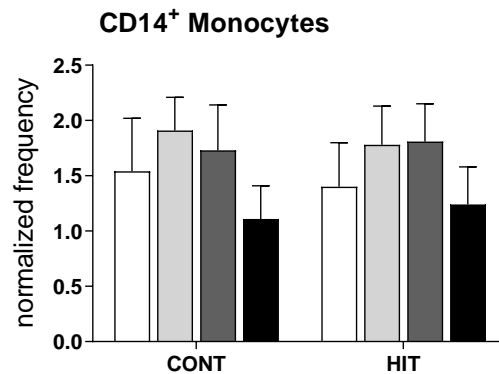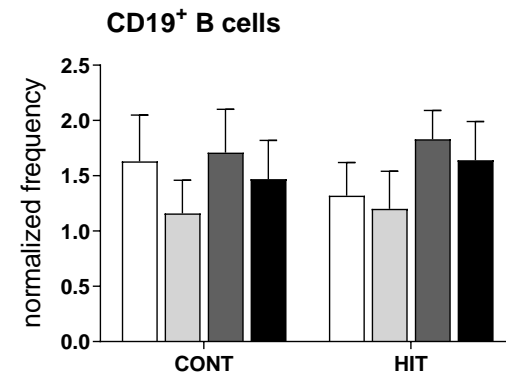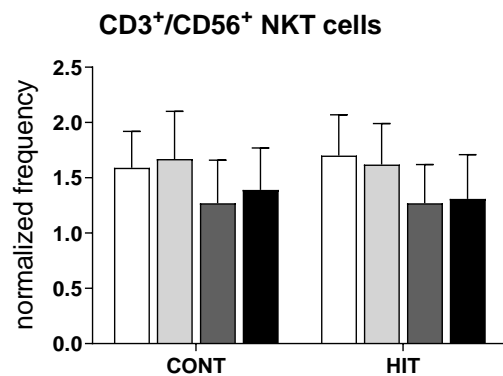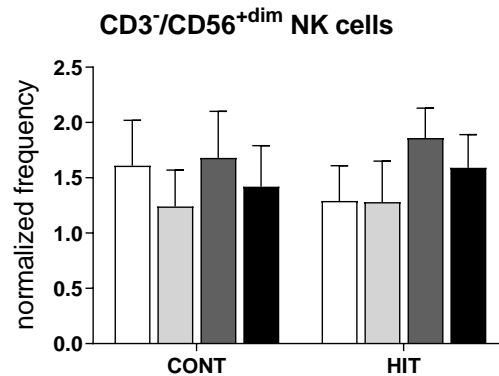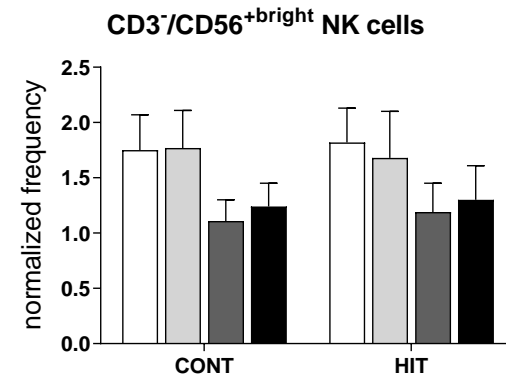

Supplement: Supplementary file 3 — Additional file 3. Changes on standard blood counts and the cellular immune status after continuous and interval exercise. Peripheral blood samples of healthy donors (n = 12) were analysed by flow cytometry at different time points before and after a single 30 min continuous (CONT) or interval (HIT) exercise (before, directly after, 1 h after and 24 h after exercise). Determined frequencies of CD45+ leukocytes, CD14+ monocytes, CD19+ B cells, CD3+/CD56+ NKT cells, and CD3−/CD56+ NK cells with their bright and dim subsets are displayed as normalized frequencies. Data are mean ± SD. [file 12967_2020_2301_MOESM3_ESM.pdf]

A

# Frequency

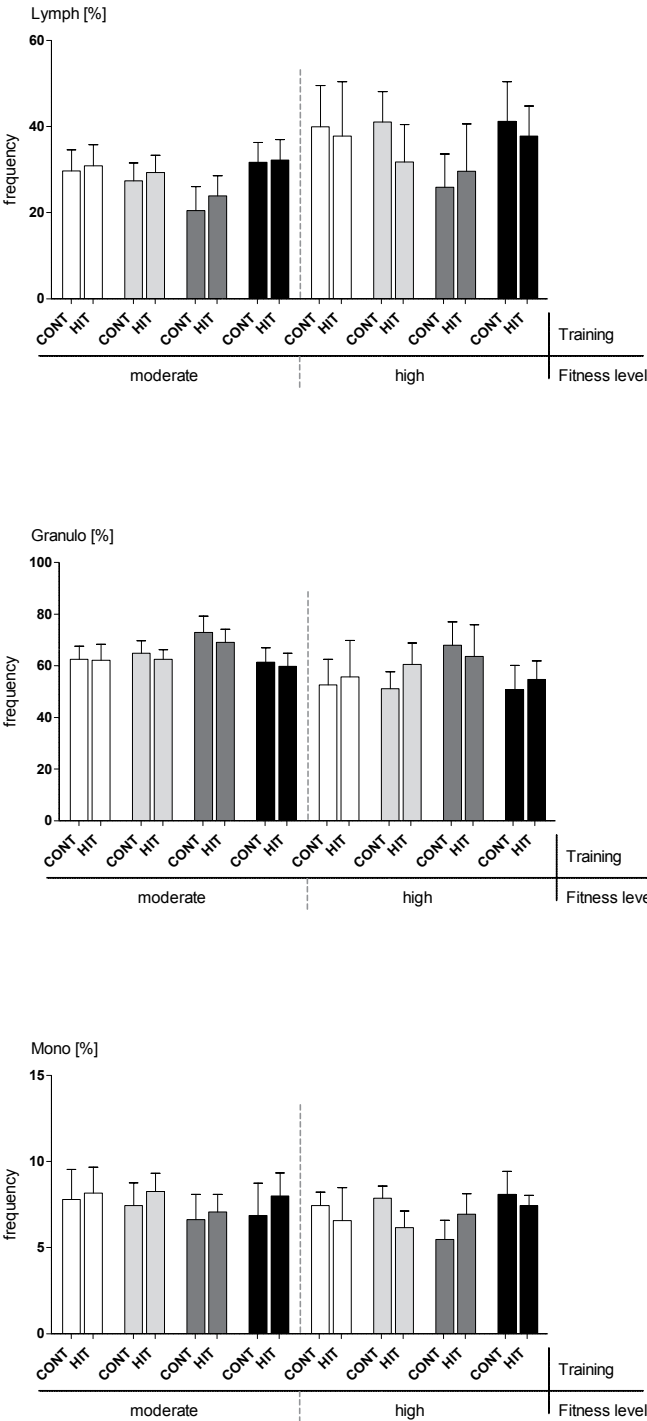

B

# Absolute count

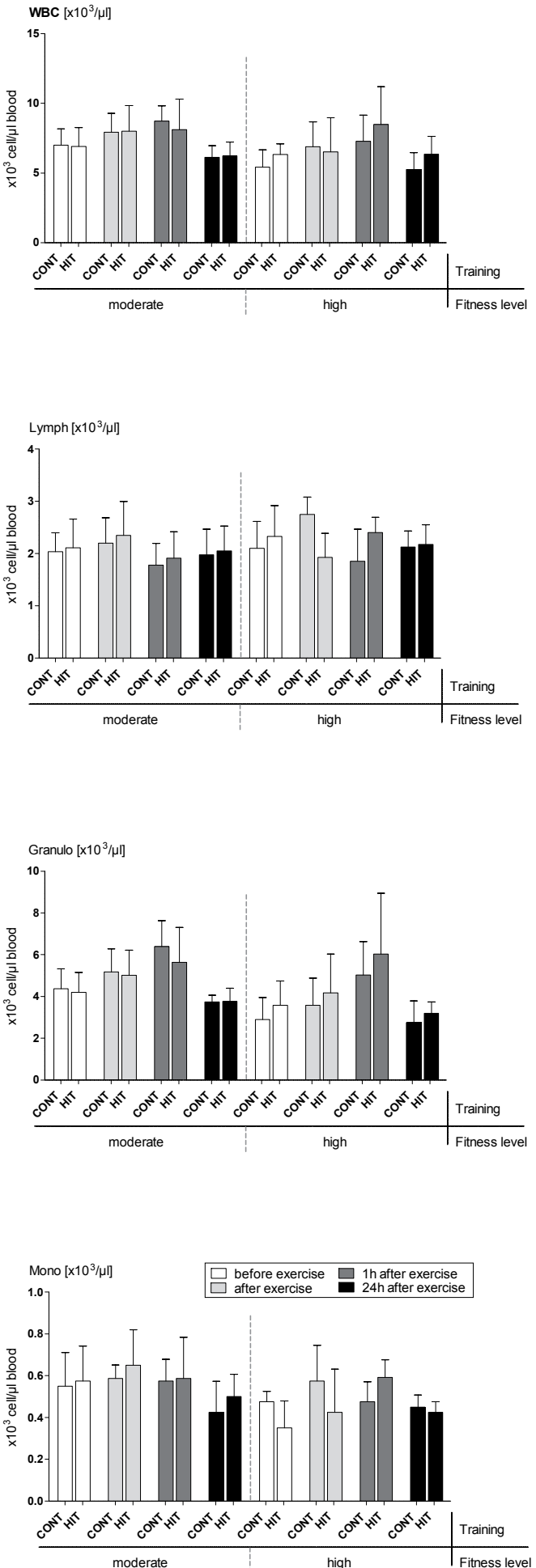

Supplement: Supplementary file 4 — Additional file 4. Changes in standard blood counts after continuous and interval exercise differentiating between moderately and highly fit donors. Peripheral blood samples of healthy donors (n = 12) were analysed using a haemocytometer at different time points before and after a single 30 min continuous (CONT) or interval (HIT) exercise (before, directly after, 1 h after and 24 h after exercise). Donors were grouped according to their fitness level (moderate, n = 8, and high, n = 4, tested with the International Physical Activity Questionnaire, IPAQ). (A) Lymphocyte, granulocyte, and monocyte frequency is shown as percentage of the total leukocyte count. (B) White blood cell (WBC), lymphocyte, granulocyte, and monocyte counts are shown as absolute counts (×103/µl blood). Data are shown as mean ± SD. [file 12967_2020_2301_MOESM4_ESM.pdf]

**A**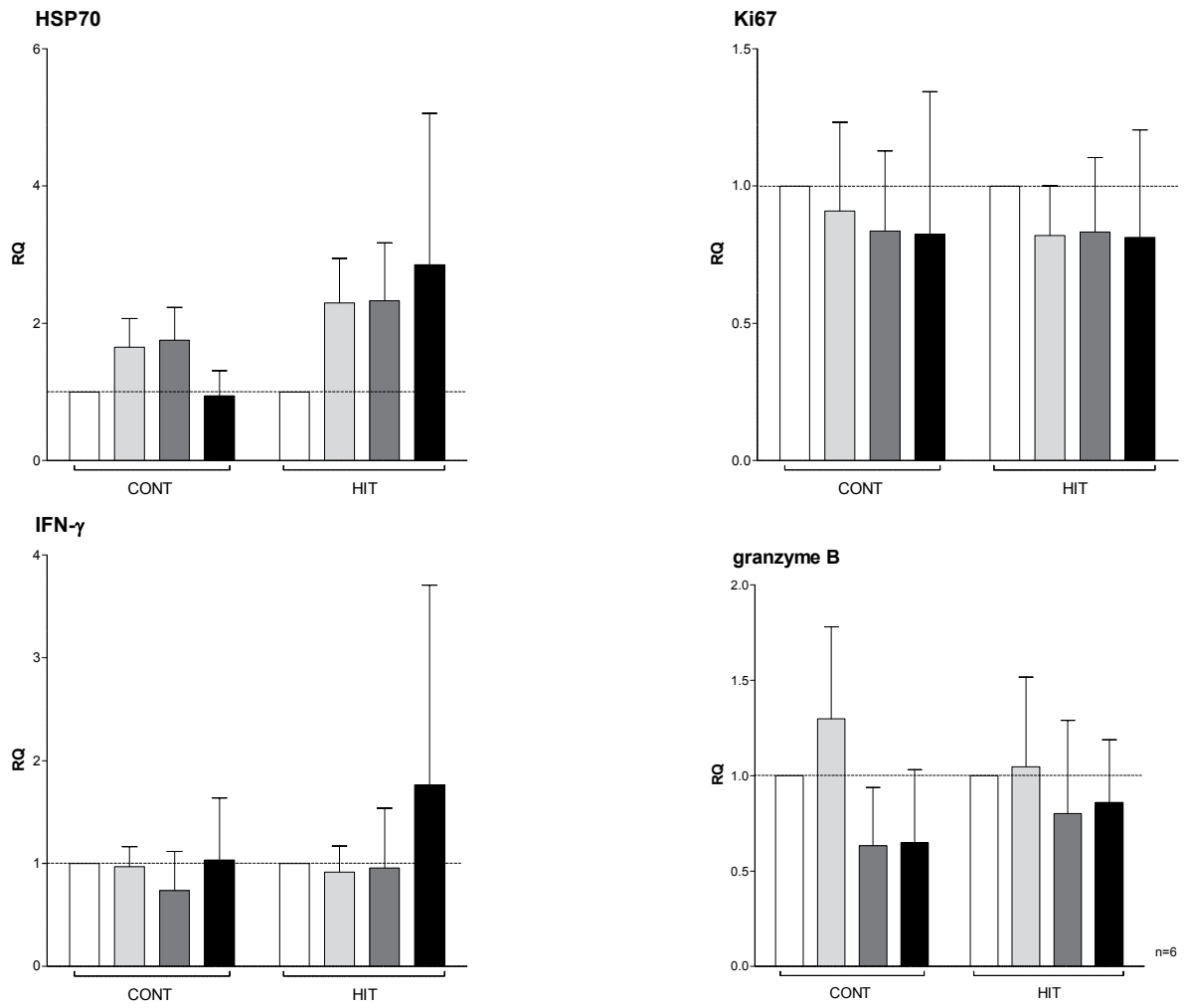**B**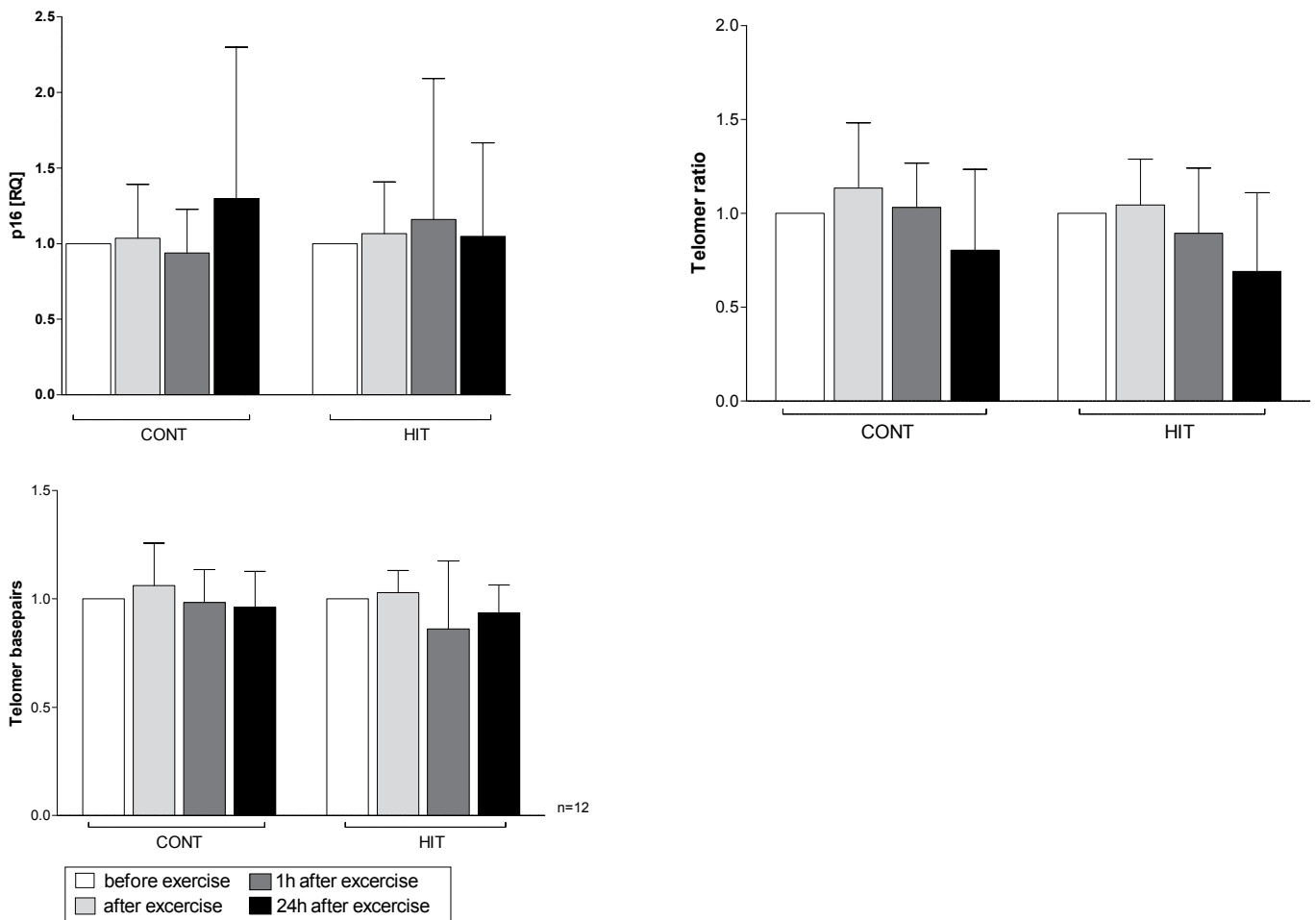

Supplement: Supplementary file 5 — Additional file 5. Effects of a single continuous and interval exercise on stress, proliferation markers, T-cell cytotoxicity and parameters of cellular age. Peripheral blood samples of healthy donors (n = 12) were analysed at different time points before and after a single 30 min continuous (CONT) or interval (HIT) exercise (before, directly after, 1 h after and 24 h after exercise) using real-time PCR for quantification of (A) HSP70- and Ki67-, IFN-γ-, and granzyme B-mRNA levels. Constitutively expressed GAPDH gene was used as the reference standard for normalization of mRNA levels. RQ values were calculated by the delta–delta CT method. (B) Blood samples were further analysed regarding p16 levels, telomere ratio and telomere base pairs. Results are displayed as RQ, with the value from before exercise being the base line, and shown as mean ± SD. [file 12967_2020_2301_MOESM5_ESM.pdf]

**A**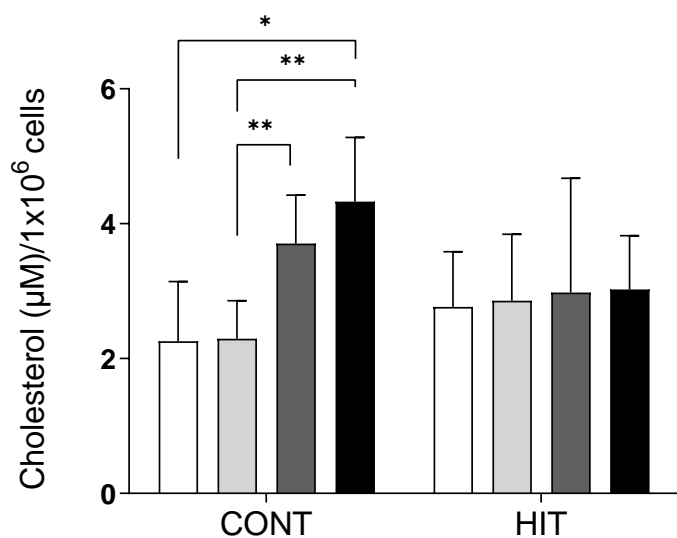**B**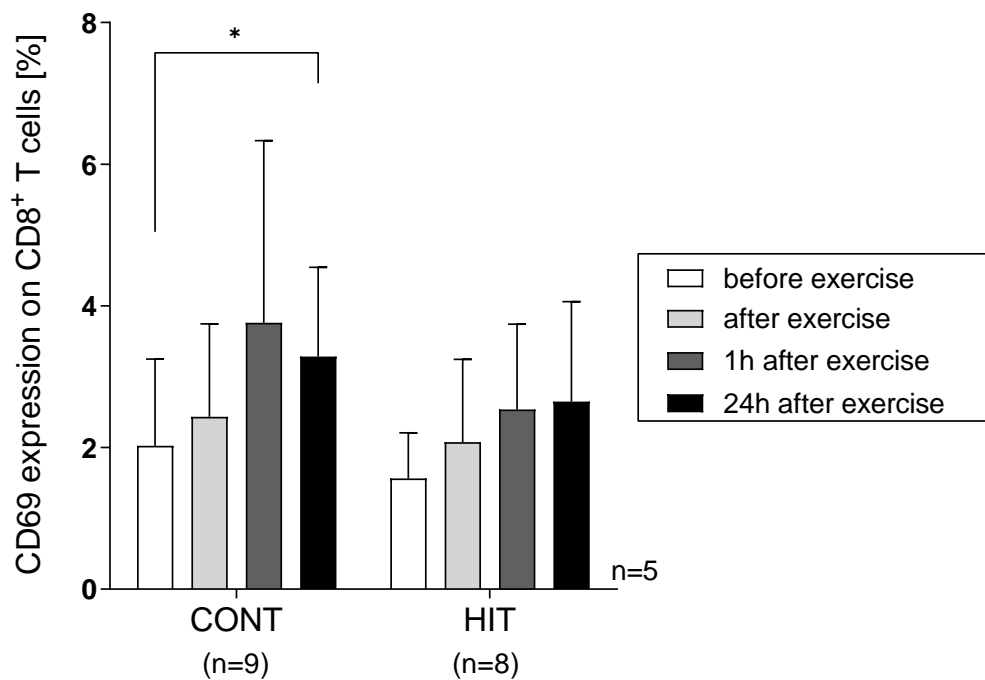

Supplement: Supplementary file 7 — Additional file 7. Effects of a single continuous and interval exercise on CD8+ T-cell cholesterol and activation levels in a donor-related setting. Peripheral blood samples of healthy donors (n = 5) were analysed at different time points before and after a single 30 min continuous (CONT) or interval (HIT) exercise (before, directly after, 1 h after and 24 h after exercise) to (A) quantify the level of cholesterol (µM)/1 × 106 cells (Amplex Red Cholesterol Assay Kit) and (B) to obtain the expression of CD69 on CD8+ T cells (flow cytometry). Data are shown in a donor-related setting as mean ± SD. Statistically significant difference is indicated by (*p < 0.05) and (**p < 0.01). [file 12967_2020_2301_MOESM7_ESM.pdf]
