# Supplementary material for: High-intensity interval training in allogeneic adoptive T-cell immunotherapy – a big HIT?
Source: J Transl Med. 2020 Apr 1;18:148. doi: 10.1186/s12967-020-02301-3 (PMC7114817; doi:10.1186/s12967-020-02301-3)
Supplement: Supplementary file 6 — Additional file 6. Impact of a single continuous and interval exercise on antigen-specific T-cell responses. Peripheral blood samples of healthy donors (n = 12) were analysed separately with the two-sample t approach adjusting for period at different time points before and after a single 30 min continuous (CONT) or interval (HIT) exercise (before, directly after, 1 h after and 24 h after exercise). Isolated PBMCs were stimulated overnight with CMV-, EBV- and AdV-specific peptide pools (CMV pp65, CMV IE1, EBV EBNA1, and EBV Consensus, AdV5 Hexon, and AdV5 Penton) and frequencies of functional-active virus-specific T cells were determined by IFN-γ EliSpot assay as spots per 1000 CD3+ T cells [file 12967_2020_2301_MOESM6_ESM.docx]

**Additional File 6**

| **Antigen** | **Sample time** | **Statistical parameter**  [regarding spots per 1000 CD3^+^ T cells] | | |  |
| --- | --- | --- | --- | --- | --- |
|  |  |  |  |  |  |
|  |  | **Effect** (HIT minus CONT) | **95% Confidence Interval** | **p-value** |  |
| **CMV pp65** | before exercise | 0.002594 | -0.03226, 0.03745 | 0.8716 |  |
|  | after exercise | -0.05198 | -0.1545, 0.05051 | 0.2848 |  |
|  | 1h after exercise | 0.04303 | -0.02774, 0.1138 | 0.2053 |  |
|  | 24h after exercise | 0.1306 | -0.02488, 0.2861 | 0.0908 |  |
| **CMV IE1** | before exercise | 0.04938 | -0.06628, 0.1650 | 0.3639 |  |
|  | after exercise | -0.04108 | -0.1253, 0.04316 | 0.3027 |  |
|  | 1h after exercise | 0.01217 | -0.01731, 0.04164 | 0.3795 |  |
|  | 24h after exercise | 0.1409 | -0.1223, 0.4041 | 0.2605 |  |
| **EBV EBNA1** | before exercise | 0.008649 | -0.1110, 0.1283 | 0.8752 |  |
|  | after exercise | -0.01478 | -0.04351, 0.01394 | 0.2781 |  |
|  | 1h after exercise | 0.002388 | -0.02595, 0.03072 | 0.8548 |  |
|  | 24h after exercise | 0.06227 | -0.00576, 0.1303 | 0.0687 |  |
| **EBV Consensus** | before exercise | 0.06027 | -0.1259, 0.2464 | 0.4872 |  |
|  | after exercise | -0.02508 | -0.1257, 0.07554 | 0.5909 |  |
|  | 1h after exercise | -0.01993 | -0.1690, 0.1291 | 0.7718 |  |
|  | 24h after exercise | 0.247 | 0.08776, 0.4062 | 0.0062 |  |
| **AdV5 Hexon** | before exercise | -0.04956 | -0.2210, 0.1219 | 0.534 |  |
|  | after exercise | -0.09857 | -0.2443, 0.04718 | 0.1628 |  |
|  | 1h after exercise | 0.008507 | -0.1781, 0.1951 | 0.9211 |  |
|  | 24h after exercise | 0.4272 | -0.01983, 0.8742 | 0.0591 |  |
| **AdV5 Penton** | before exercise | -0.00751 | -0.08889, 0.07388 | 0.8413 |  |
|  | after exercise | -0.04522 | -0.09533, 0.004891 | 0.0721 |  |
|  | 1h after exercise | -0.00073 | -0.06053, 0.05908 | 0.9789 |  |
|  | 24h after exercise | 0.1636 | 0.04288, 0.2842 | 0.0129 |  |
|  |  |  |  |  |  |
| Peripheral blood samples of healthy donors (n=12) were analysed separately with the two-sample t approach adjusting for period at different time points before and after a single 30 minute continuous (CONT) or interval (HIT) exercise (before, directly after, 1h after and 24h after exercise). Isolated PBMCs were stimulated over night with CMV-, EBV- and AdV-specific peptide pools (CMV pp65, CMV IE1, EBV EBNA1, and EBV Consensus, AdV5 Hexon, and AdV5 Penton) and frequencies of functional-active virus-specific T cells were determined by IFN-γ EliSpot assay as spots per 1000 CD3^+^ T cells. | | | | |  |
